# The ISCB competency framework v. 3: a revised and extended standard for bioinformatics education and training

**DOI:** 10.1093/bioadv/vbae166

**Published:** 2024-11-18

**Authors:** Cath Brooksbank, Michelle D Brazas, Nicola Mulder, Russell Schwartz, Verena Ras, Sarah L Morgan, Marta Lloret Llinares, Patricia Carvajal López, Lee Larcombe, Amel Ghouila, Tom Hancocks, Venkata Satagopam, Javier De Las Rivas, Gaston Mazandu, Bruno Gaeta

**Affiliations:** EMBL’s European Bioinformatics Institute, Wellcome Genome Campus, Cambridge CB10 1SD, United Kingdom; Ontario Institute for Cancer Research, Toronto, ON M5G 0A3, Canada; Computational Biology Division, Department of Integrative Biomedical Sciences, Faculty of Health Sciences, University of Cape Town, Cape Town, 7925, South Africa; Department of Biological Sciences and Computational Biology Department, Carnegie Mellon University, Pittsburgh, PA 15213, United States; Computational Biology Division, Department of Integrative Biomedical Sciences, Faculty of Health Sciences, University of Cape Town, Cape Town, 7925, South Africa; EMBL’s European Bioinformatics Institute, Wellcome Genome Campus, Cambridge CB10 1SD, United Kingdom; EMBL’s European Bioinformatics Institute, Wellcome Genome Campus, Cambridge CB10 1SD, United Kingdom; EMBL’s European Bioinformatics Institute, Wellcome Genome Campus, Cambridge CB10 1SD, United Kingdom; Nexastem Ltd, St Neots, Cambridgeshire PE19 6AD, United Kingdom; Computational Biology Division, Department of Integrative Biomedical Sciences, Faculty of Health Sciences, University of Cape Town, Cape Town, 7925, South Africa; EMBL’s European Bioinformatics Institute, Wellcome Genome Campus, Cambridge CB10 1SD, United Kingdom; Luxembourg Centre For Systems Biomedicine, University of Luxembourg, Esch-sur-Alzette L-4365, Luxembourg; Cancer Research Center (CiC-IBMCC), Consejo Superior de Investigaciones Científicas (CSIC) and University of Salamanca (USAL), Salamanca 37007, Spain; Division of Human Genetics, Department of Pathology, Faculty of Health Sciences, University of Cape Town, Cape Town, 7925, South Africa; School of Computer Science and Engineering, UNSW Sydney, Sydney, NSW 2052, Australia

## Abstract

**Motivation:**

Developing competency in the broad area of bioinformatics is challenging globally, owing to the breadth of the field and the diversity of its audiences for education and training. Course design can be facilitated by the use of a competency framework—a set of competency requirements that define the knowledge, skills and attitudes needed by individuals in (or aspiring to be in) a particular profession or role. These competency requirements can help to define curricula as they can inform both the content and level to which competency needs to be developed. The International Society for Computational Biology (ISCB) developed a list of bioinformatics competencies in 2014, and these have undergone several rounds of improvement. In consultation with a broad bioinformatics training community, these have now been further refined and extended to include knowledge skills and attitudes, and mappings to previous and other existing competency frameworks.

**Results:**

Here, we present version 3 of the ISCB competency framework. We describe how it was developed and how to access it, as well as providing some examples of how it has been used.

**Availability and implementation:**

The framework is openly accessible at https://competency.ebi.ac.uk/framework/iscb/3.0/competencies.

## 1 Introduction

Bioinformatics and computational biology are diverse fields spanning a number of traditional subject areas, including computer science, biochemistry, microbiology, genetics and, increasingly, mathematics and statistics. More recently, as data size and complexity have grown, there has been an increased demand for the application of data science techniques to life sciences research. This is accompanied by an increased need for data science and computational biology skills across a broad range of professions, from classical bench research to medicine, agronomy, and conservation. Researchers who need to apply computational biology competencies range from wet-lab scientists or medical professionals needing to run basic analyses and interpret results, via computer scientists who develop novel algorithms and containerized workflows, to data scientists who model complex datasets (structured and unstructured) and design predictive analytics. Employers seeking to recruit someone in the field often struggle to assess how competent applicants are for the role and, conversely, employees struggle to evaluate their skills gaps when exploring potential career paths. Bioinformatics trainers and educators also face many challenges in developing the necessary competencies through their learning interventions. Course participants often start with different levels of competency and can have high expectations of what they will be able to achieve after training. A competency framework provides a standard and tangible measure of the output and outcomes of a learning intervention. It can be used during the design or review of a curriculum and its content. Defined competency requirements can also help to conceptualize standards of excellence for different organizational roles and potential opportunities, improving the ability to match needs to the most appropriate employees.

Competency is an observable and measurable characteristic of a professional. It incorporates the knowledge, skills, and attitudes (KSAs) that the person needs to fulfil in a specific context (e.g. to gain a qualification, or to perform adequately in a job role). A competency framework defines the set of competencies required in a given context or set of contexts. These concepts are explained in more detail in a Competency Guidelines Document (Schwartz *et al.* 2021) developed by some of the authors of this paper.

The International Society for Computational Biology (ISCB) developed a set of core competencies to guide bioinformatics curriculum development in 2014 (Welch *et al.* 2014), based on groundwork prepared by the ISCB Education Committee over the previous decade (Welch *et al.* 2012). These were evaluated and refined as they were applied in different contexts (Welch *et al.* 2016). The ISCB Competency Framework uses personas—defined characters (which are fictional but based on a melange of factual roles) with a specific career profile. The framework defines the level of competency required by each career profile in an agreed set of areas spanning bioscience, data science, computer science and professional conduct. Over time, through engagement with a variety of stakeholders committed to bioinformatics education and career development (Box 1), the persona concept was adapted to encompass professionals in a wider range of career roles; some competencies were refined accordingly, and levels of competency appropriate to each role were agreed and mapped to Bloom’s taxonomy (Anderson and Krathwohl 2001). Bloom’s taxonomy provides a common language for educators to discuss and exchange learning and assessment methods. It is used to assess learning on a variety of cognitive levels, from retention of facts through application and synthesis to the creation of original work. Versions 2 and 3 of the ISCB Competency Framework use Bloom’s taxonomy to define the expertise level required in a given role; e.g. a bioinformatics software developer needs a high Bloom’s level of competency G3 (Contribute effectively to the design and development of user-centric bioinformatics tools and resources), but a basic working knowledge of competency A3 (“Work at depth in at least one technical area aligned with the life sciences”); by contrast, a discovery biologist might be revealing new things about biological mechanism and therefore at the top of the Bloom’s scale for competency A3, yet need only enough understanding of software engineering to have a meaningful conversation with a specialist in this field. This resulted in the release of an improved version of the ISCB competency framework (version 2.0), which has been applied to several use cases in short training course development and in the development or assessment of degree programmes (Mulder *et al.* 2018).

Since the release of the ISCB competency framework version 2.0 (Mulder *et al.* 2018), several workshops have been held at various conferences and education events to seek input on its structure and to support trainers and educators to use the framework to develop or update courses and curricula. Through these, additional challenges were identified, including the scope of the roles represented, lack of clarity about the knowledge, skills, and attitudes associated with each competency, and the need for practical guidance on how to use the framework to design a course or curriculum. This led us to refine the framework significantly, including the addition of knowledge, skills, and attitudes (KSAs) associated with each competency. We continue to develop new career profiles as bioinformatics competency requirements seep into an ever-widening array of roles. Here we present version 3.0 of the ISCB Competency Framework, including mappings to previous versions for those who have worked extensively with them. All three versions of the ISCB competency framework are openly available at https://competency.ebi.ac.uk/framework/iscb/3.0/competencies.

## 2 Methods

The Curriculum Task Force of the ISCB Education Committee, together with a diverse panel of bioinformatics domain experts and users (Box 1), developed version 3.0 of the ISCB competency framework using an iterative consultation process. This work was started at the first Global Bioinformatics Education Summit, held in Cape Town, South Africa, in May 2019, continued at ISMB/ECCB 2019 in Basel, Switzerland, and then at the second Bioinformatics Education Summit, held virtually in 2020 and hosted by EMBL-EBI. Between these meetings, a small task force of bioinformatics trainers and training programme managers discussed and integrated suggestions and updates through online meetings. The 2019 meetings focused on brainstorming and roughly drafting the KSAs, drawing on several related frameworks for inspiration and using the computational biology expertise of those convened to rethink and update the competency requirements.

At the Cape Town Summit, participants split into breakout groups, each with a subset of competencies to work on, and used a worksheet per competency to capture their consensus on the prerequisite attributes (knowledge, skills, and attitudes) required. Their work was presented back in plenary and discussed among all participants. The output from this meeting was then reviewed by a focus group at the Education session of ISMB/ECCB 2019, and further suggestions for improvement were incorporated.

Work at the 2020 Bioinformatics Education Summit focused on consolidating the vast amount of content that we had accumulated, and on standardizing the syntax of the framework. Each member of the task force volunteered to focus on a subset of the competencies, review KSAs, and consolidate them into a manageable number of attributes associated with each competency. Work continued beyond the summit meeting, again with a small task force, this time dedicated to finalising the content of the framework.

### 2.1 Viewing the framework as a minimum standard

One important decision made at the 2020 Bioinformatics Education Summit simplified our task: we agreed that an individual is considered to fulfil a competency if they demonstrate all of the knowledge, skills, and effective attitudes listed for that competency to the level required of a given persona or career profile. This decision was important philosophically because it transformed the framework into a minimum standard. It was also important practically because it helped us to decide which KSAs to delete, and to reach a consensus on which competencies needed to be merged or split. When we then came to update the levels required for each of the competencies previously defined in version 2.0 of the framework (Mulder *et al.* 2018), we could define a single minimum threshold Bloom’s level required for each competency. An individual must be minimally competent at the defined Bloom’s level—knowledge (Bloom’s level 1), comprehension (2), application (3), analysis (4), synthesis (5), and evaluation (Bloom’s level 6)—for a given competency (Anderson and Krathwohl 2001). For example, a bioinformatics software developer would need all the KSAs listed in competency A3 (“Work at depth in at least one technical area aligned with the life sciences”) at Bloom’s level 2 (understand), but all the KSAs listed in competency G3 (“Contribute effectively to the design and development of user-centric bioinformatics tools and resources”) at Bloom’s level 6 (evaluate). Using this updated competency framework, bioinformatics career profiles were then remapped to version 3.0 competencies to define the minimum competency requirement for each of the personas/career profiles (Table 1). The diversity of career profiles represented in the taskforce itself has enabled us to cross-check our assumptions and come to a consensus on competency requirements; typically each career profile has been assessed by five or more individuals in roles related to that profile.

**Table 1.** The ISCB Competency Framework version 3.0 and minimum competency levels (Bloom’s) required for each bioinformatics career profile.

Domain	Competency	Competency description	Physician V3	Laboratory technician V3	Ethicist V3	Biocurator V3	Discovery biologist V3	Molecular life science educator V3	Bioinformatics researcher V3	Core facility scientist V3	Bioinformatician in an academic or Research Infrastructure support role V3	Bioinformatics software developer/software engineer V3
Bioscience	A3	Work at depth in at least one technical area aligned with the life sciences.	Application	Application	Evaluation	Application	Evaluation	Analysis	Evaluation	Evaluation	Comprehension	Comprehension
	B3	Prepare life science data for computational analysis	Knowledge	Knowledge	Knowledge	Knowledge	Evaluation	Comprehension	Evaluation	Evaluation	Comprehension	Comprehension
	C3	Have a positive impact on scientific discovery through bioinformatics	Application	Comprehension	Knowledge	Comprehension	Application	Analysis	Synthesis	Application	Application	Application
Data science	D3	Use data science methods suitable for the size and complexity of the data	Knowledge	Knowledge	Knowledge	Comprehension	Application	Evaluation	Synthesis	Application	Application	Application
	E3	Manage own and others’ data according to community standards and principles	Comprehension	Comprehension	Knowledge	Application	Application	Comprehension	Application	Evaluation	Analysis	Application
	F3	Make appropriate use of bioinformatics tools and resources	Comprehension	Knowledge	Knowledge	Application	Application	Evaluation	Synthesis	Application	Evaluation	Evaluation
Computer science	G3	Contribute effectively to the design and development of user-centric bioinformatics tools and resources	N/A	Knowledge	N/A	Application	Knowledge	Knowledge	Comprehension	Evaluation	Evaluation	Evaluation
	H3	Make appropriate and efficient use of scripting and programming languages	N/A	Knowledge	N/A	Comprehension	Knowledge	Knowledge	Application	Evaluation	Analysis	Evaluation
	I3	Construct, manage and maintain bioinformatics computing infrastructure of varying complexity	N/A	N/A	N/A	Knowledge	N/A	N/A	Synthesis	Evaluation	Analysis	Evaluation
Professional conduct	J3	Comply with professional, ethical, legal and social standards and codes of conduct relevant to computational biology	Application	Application	Evaluation	Analysis	Application	Application	Application	Application	Comprehension	Comprehension
	K3	Communicate meaningfully with a range of audiences - within and beyond your profession	Application	Application	Application	Application	Application	Application	Application	Application	Application	Application
	L3	Work effectively in teams to accomplish a common goal	Application	Analysis	Application	Analysis	Application	Application	Application	Application	Application	Application
	M3	Engage in continuing professional development in bioinformatics	Application	Application	Application	Application	Application	Application	Application	Application	Application	Application

### 2.2 Agreeing on syntax

We have used consistent syntax throughout version 3.0 of the framework:

- All competencies (A-M) in version 3.0 are labelled with 3 (A3, B3, etc.) to distinguish them from version 2.0 competencies (labelled A2, B2, etc.).
- Each attribute is labelled with an identifier that describes whether it is an area of knowledge (K), a skill (S), an effective attitude (A), or an ineffective attitude (N), and has an identifier that relates it to the competency and framework version with which it is associated (e.g. knowledge attributes for competency B3 are labelled with “KB3-1, KB3-2, etc.” where K indicates this is a knowledge attribute), B indicates its parent is competency B—Prepare life science data for computational analysis—and 3 indicates the version of the competency framework to which it belongs (see Table 2).
- Competencies and their attributes are now active statements:
- Each competency can be prefaced with the phrase “A competent bioinformatics professional will…”: e.g. A competent bioinformatics professional will (A3) “Work at depth in at least one technical area aligned with the life sciences.”
- For a knowledge attribute: A competent bioinformatics professional knows… e.g. A competent bioinformatics professional knows (KA3-1) “The central dogma, general biological concepts and how they relate to each other.”
- For a skill: A competent bioinformatics professional… e.g. A competent bioinformatics professional (SA3-1) “Differentiates between biological and non-biological entities.”
- For an effective attitude: A competent bioinformatics professional… e.g. A competent bioinformatics professional (AA3-2) “Integrates ideas from discipline-specific communities.”
- For an ineffective attitude: An ineffective bioinformatics professional… e.g. An ineffective bioinformatics professional (NC3-1) “Has a negligent attitude towards data quality/integrity.”

**Table 2.** Example competency.^a^

B3: Prepare life science data for computational analysis	
Knowledge KB3-1: Details of omic-scale/big-data-driven life science core platform technologies KB3-2: Applications and limitations of the technologies relevant to the chosen field KB3-3: Sources of errors in data generated by the relevant technologies KB3-4: To collect experimental data in relevant formats that are suitable for subsequent computational analysis	Skill SB3-1: Recognises and critically reviews the format, scope and limitations of different biological data-generating platforms SB3-2: Applies technology and methodology considering the experimental material available and the objective of the experiment SB3-3: Constructs data management plans for projects, including consideration of data curation SB3-4: Documents the data-generation methodology to optimise reproducibility
Effective attitudes AB3-1: Ensures that data are generated in compliance with legal, ethical and commercial regulations relevant to the context AB3-2: Recognises limitations in data-generation technologies, stays up to date with development of new ones, and consults experts when required AB3-3: Demonstrates awareness of the need for data management and documentation AB3-4: Manages relationship between the data generator and the data recipient effectively	Ineffective attitudes NB3-1: Has a negligent attitude towards data quality/integrity
Competency derived from: ISCB n1 , ISCB C2, UKNOS COGBIO-03, UKNOS COGLS322, UK K4, UA S25	

a An example competency from the version 3.0 ISCB Competency Framework showing the Knowledge (K), Skill (S) and Attitudes (A) required of the competency. Table also shows which other competency frameworks were used to create these KSAs. A detailed mapping file with additional information about the provenance of mappings between different versions of the framework is available in the Supplementary Material.

### 2.3 Incorporating new themes

Where new fields of importance to computational biology have emerged (or where pre-existing fields have assumed new relevance, such as artificial intelligence and machine learning), we have updated the framework to incorporate these fields. For example, competency E2 (Statistical research methods in the context of molecular biology, genomics, medical, and population genetics research) has been broadened in scope to include all forms of data science, from classical statistics through to artificial intelligence and machine learning, and is now D3: “Use data science methods suitable for the size and complexity of the data.” We also created a new competency, E3 (Manage own and others’ data according to community standards and principles), intended to capture the need for bioinformatics professionals to demonstrate data management competency and to encourage the adoption of FAIR principles (Wilkinson *et al.* 2016) in the field.

### 2.4 Mapping to other frameworks

Version 2 of the ISCB Competency Framework has been widely used as the basis of other competency-based projects. Especially significant among these were two UK-based projects:

- Development of a National Occupation Standard to describe a “Bioinformatics Scientist” (https://www.ukstandards.org.uk/NOS-Finder#k=bioinformatics);
- Creation of a Level 7 Degree Apprenticeship standard for “Bioinformatics Scientist” (Anon 2018).

In the UK, apprenticeships allow individuals to qualify for a specific profession through paid work. Whilst classically used to train for vocational roles rather than those requiring an academic qualification, the UK introduced higher-level apprenticeships in 2010, allowing individuals to reach up to master’s level through an apprenticeship. Master’s level bioinformatics apprenticeships were introduced in 2019. Both standards listed above can be used by employers to develop or define role descriptions, and the apprenticeship standard is used by universities to offer level 7 degree-based programmes for employers to train apprentices. This work, carried out by an industry- and employer-led group, involved developing a set of competencies with associated KSAs, as well as clearly defined endpoint assessment criteria (Anon 2018) to evaluate competency on completion of the apprenticeship. Version 2 of The ISCB competency framework heavily influenced the initial competencies used in the development of these standards, and employer-relevant KSAs were added. In turn, when we came to add KSAs to create version 3 of the ISCB framework, we turned to those developed by the UK standards for inspiration and incorporated much of their work.

**Figure 1.**
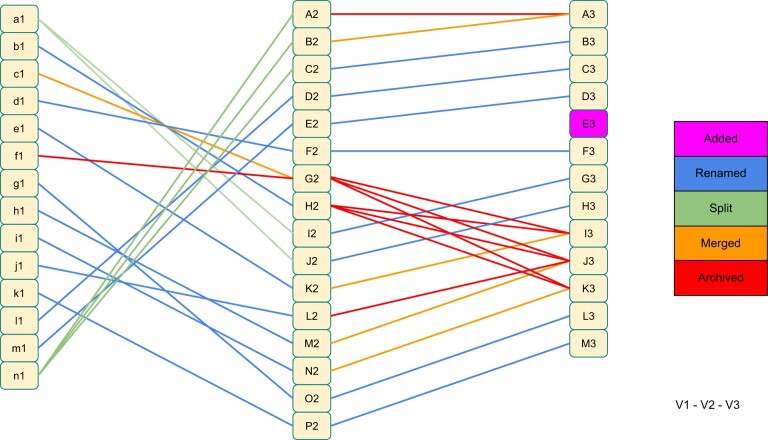
Mapping to previous versions. Summary diagram showing the relationship of each competency to its predecessors across versions 1.0, 2.0, and 3.0 of the ISCB Competency Framework. The dataset from which this mapping is derived is provided in the Supplementary Material, together with mappings to other frameworks. This information was also used to populate the interactive version of the framework at https://competency.ebi.ac.uk/framework/iscb/3.0/competencies.

Similarly, when version 1.0 of the ISCB framework was in development and we incorporated competencies required by bioinformatics engineers, we turned to the Engineers Australia Stage 1 Competency Standard for inspiration. Whilst Engineers Australia do not use identical terminology, many of the competencies in this standard—especially the attitudinal/behavioural competencies—shaped the ISCB version 3.0 framework. An example of how these mappings look in the competency hub is shown in Table 2. A full table of mappings is available in Supplementary Material S1 and is represented graphically in Fig. 1.

Finally, part of the ISCB framework (primarily competencies J3–M3) defines professional competencies rather than scientific or technical competencies. Here, we were influenced by two projects, each of which has defined a competency framework for its own learners. RItrain (Research Infrastructure Training Programme) developed a framework for research infrastructure managers, and CORBEL (Coordinated Research Infrastructures Building Enduring Life-science) for technical operators of research infrastructure. Both used KSAs and we have borrowed from these in the ISCB professional competencies.

**Figure 2.**
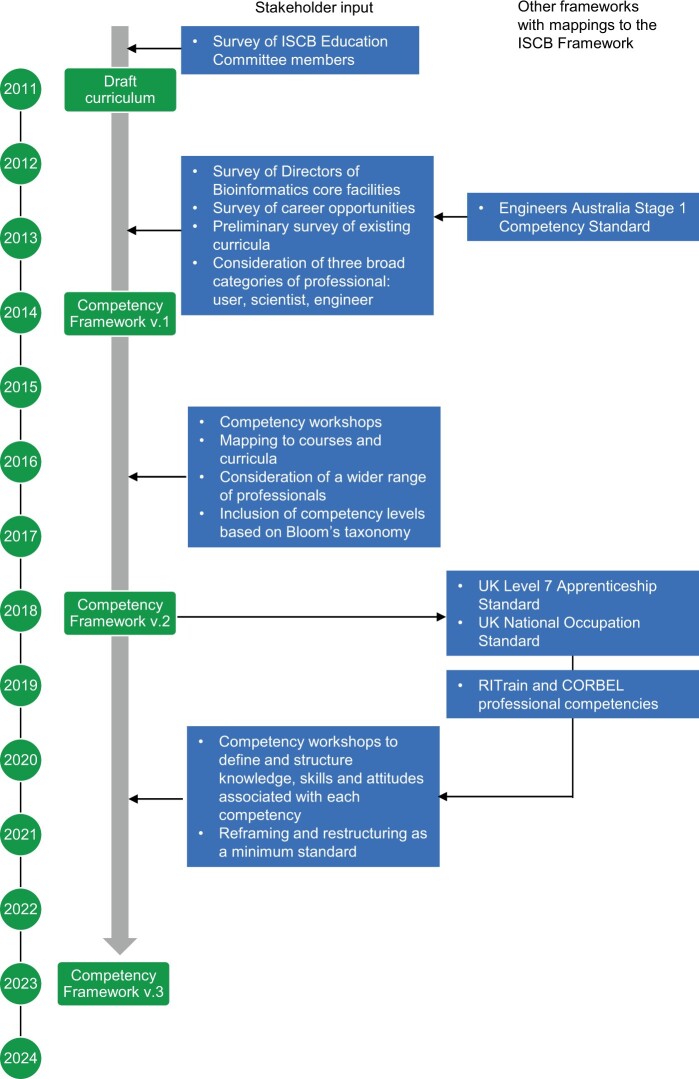
Evolution of the ISCB competency framework. This timeline summarizes the key inputs to the ISCB competency framework at different stages of its development (boxes with arrows). In the case of the UK level 7 apprenticeship standard and National Occupation Standard, the relationship is cyclical: version 2 of the ISCB Competency framework informed development of the UK standards; the knowledge, skills and attitudes developed by standards task forces were then used to inform version 3 of the ISCB framework. Mappings to all the frameworks listed here are detailed in the Supplementary Material.

We have made our mappings transparent by publishing all three versions of the ISCB competency framework, together with mappings between versions and to other frameworks, in the EMBL-EBI Competency hub (https://competency.ebi.ac.uk/framework/iscb/3.0/competencies). Clicking on any competency opens the details page, where the KSAs are listed. This page also has a “competency derived from” section; where relevant, the reference number relating to the source competency or KSA is provided in this section (see Fig. 2 and Supplementary Material S1). The following nomenclature is used for the related competency frameworks:

1. EA: Engineers Australia (https://www.engineersaustralia.org.au/).
2. UA: UK level 7 apprenticeship standard (https://www.instituteforapprenticeships.org/apprenticeship-standards).
3. UKNOS: UK National Occupation Standard (https://www.ukstandards.org.uk/NOS-Finder).
4. RI: RItrain (http://ritrain.eu/; https://competency.ebi.ac.uk/framework/ritrain/1.0).
5. CO: CORBEL (https://www.corbel-project.eu/home.html; https://competency.ebi.ac.uk/framework/corbel/1.1).

We also considered mapping to the Mastery Rubric (Tractenberg *et al.* 2019), a tool developed to support bioinformatics curriculum development. Either, or both, tools can be used by educators to support the development of bioinformatics curricula, or by individuals to self-assess and guide personal and professional development. The 12 KSAs that comprise the Mastery Rubric can all readily be identified in the ISCB competency framework. Our decision to focus on a wide range of specialist career profiles that incorporate aspects of bioinformatics without requiring mastery of all competency areas led us to conclude that mapping to the mastery rubric would not be helpful for them. Nevertheless, because of the consistent use of Bloom’s terms in both, such a mapping would be feasible and could be added to future versions of the ISCB Competency Framework if there was demand for this from the ISCB education community.

## 3 Results and discussion

### 3.1 The updated competency framework

Version 3.0 of the ISCB Competency Framework contains 13 competency statements, A3 to M3 (Table 1). These have been re-organized so that the bioscience-related competencies appear together (A3-C3), then data science (D3-F3), then computer science (G3-I3), and then professional conduct (J3-M3). Version 3.0 includes several major revisions to version 2.0 (Welch *et al.* 2016):

Firstly, competency descriptions are now all active statements. In version 3.0, each competency can be prefaced with the phrase “A competent bioinformatics professional will…”. For example, a competent bioinformatics professional will (B3) prepare life science data for computational analysis.

The second major change is the creation of component attributes for each competency. Referred to as KSAs, these components incorporate knowledge, skills, and attitudes. Attitudes are further delineated into effective and ineffective attitudes. An individual is considered to be competent in an area if they display all of the knowledge, skills, and effective attitudes at the indicative Bloom’s level (Anderson and Krathwohl 2001) for their role (see Table 2 for an example), whilst avoiding behaviours underpinned by the ineffective attitudes. Each competency was populated with enough attributes to provide evidence that an individual has acquired the competency. Opinion is divided on whether ineffective attitudes should be included in the framework; those who favour their inclusion argue that it is just as helpful to know what you should not be doing in a professional context as it is to know what you should be doing. Those who find them unhelpful for their own particular application have no obligation to use them.

Thirdly, mappings to versions 1.0 and 2.0 have been added and can be viewed by end-users by clicking on the information icon and going to the competency-specific page, which also provides mappings to relevant courses. (Table 2). Users can click through to the relevant competency in the previous version. As noted previously, many of the competencies and their attributes are derived from those in other competency frameworks so, where relevant, the reference number relating to the source competency or attribute is also provided in the “Competency derived from” section (Table 2).

### 3.2 High-level revisions within the competencies

A high-level summary of Version 3.0 of the ISCB Competency Framework is presented in Table 1. In addition to major changes to the competency framework itself, several major changes were made to individual competencies, as summarized in Fig. 1. Firstly, the two general biology competencies A2 and B2 were merged into A3 to eliminate redundancy. Competencies G2, H2, and L2 were deprecated and their concepts repositioned within another version 3.0 competency; specifically, concepts from H2 and G2 were incorporated into I3, J3, and K3, and concepts from L2 were incorporated into J3. A new competency, E3, was added to the framework to capture the need for bioinformatics professionals to demonstrate competency in data management and to encourage the adoption of FAIR principles (Wilkinson *et al.* 2016) in the field. Given these merge, addition, and archiving activities, the remaining competencies required updated numbering; specifically, C2, D2, and E2 were renumbered to B3, C3, and D3 respectively; I2, J2, and K2 were renumbered to G3, H3, and I3 respectively; and M2, O2, N2, and P2 were renumbered to J3, K3, L3, and M3 respectively.

A table of mappings, including mappings to other competency frameworks, is provided in Supplementary Material S1.

### 3.3 Using the ISCB competency framework

By providing a description of the areas of competency required, the Bloom’s level at which they’re required, and the knowledge, skills and attitudes that demonstrate each competency, Version 3 of the ISCB competency framework can be used to design new curricula, to evaluate existing ones, and to review and improve curricula by combining both processes.

The framework can also be used by individuals to assess their own competency, plan their career development and discover training opportunities to build specific competencies. Previous versions of the ISCB Competency Framework have been applied successfully to course design, evaluation, and review for courses at multiple levels as well as entire degree programmes (Welch *et al.* 2016, Mulder *et al.* 2018). To facilitate the continued application of this framework, some co-authors of this paper have written a comprehensive guidelines document (Schwartz *et al.* 2021).

### 3.4 How can course providers use the framework?

The design process for a new course or programme begins with the identification of the desired target career profile for graduates of the course/programme, and then uses the competencies and their associated attributes to formulate learning outcomes. These learning outcomes can then be used to design new courses or identify existing courses that deliver these outcomes. For example, if one was designing a course for physicians to support -omics-based clinical decision-making, one might select the Physician career profile and design learning outcomes based on the Bloom’s levels required for this role. In this case, there might also be additional competencies, linked to the intersection of bioinformatics with clinical decision-making but not common to all bioinformatics roles, that would need to be considered. In clinical settings, national variations in clinical practice come into play, e.g. and would need to be taken into account. In the UK, such a course might include training on the role of clinical bioinformaticians in supporting clinical decisions.

Similarly, the evaluation of an existing course or programme begins with taking its learning outcomes and mapping these to the competency requirements for a target career profile/persona in the ISCB Competency Framework version 3.0. The KSAs associated with each competency facilitate the mapping of learning outcomes to competencies and vice-versa, by making it easier to spot common outcomes. It is worth noting that KSAs act simply as indicators of attainment of the competency; it may not be necessary to deliver content pertaining to each KSA to support learners’ development of the competency. The KSAs need to be considered in the context of the course/programme being reviewed and the anticipated existing competency level of the course participants. The important thing is that, once a learner has completed the course, they can demonstrate all the KSAs associated with a competency to the level needed for their role.

Revision of an existing course/programme also begins by mapping the learning outcomes of the course to the competencies and their KSAs, to identify the competencies that are lacking or over-represented in the curriculum’s learning outcomes. Missing competencies can be addressed using the design process described for a new course. If space is needed to accommodate new content to address the need for missing competencies, over-represented competencies indicate which content might be removed with the least impact on overall learning outcomes.

### 3.5 Course design and evaluation case studies

Examples of application of the ISCB Competency Framework are described in detail in the Supplementary Materials (Gaëta *et al.* 2021), where links to course mappings are also provided. These include the use of the competencies for designing new courses and programmes: a new short training course (the H3ABioNet’s 16S RNA bioinformatics course), and a new university degree programme (the MSc programme offered by the Kwame Nkrumah University of Science and Technology, Ghana). The framework was also used to review and update existing courses: individual training courses (e.g. H3ABioNet’s Introduction to Bioinformatics) and university degree programme [e.g. Bachelor of Engineering (Bioinformatics Engineering) offered by the University of New South Wales, Sydney, Australia]. A summary of these approaches is provided below.

**New training course:** This use-case focuses on the development of H3ABioNet’s 16S rRNA Intermediate Bioinformatics Training (Int_BT) Course. In this case, the ISCB Competency Framework was used to facilitate the mapping of competencies and KSAs concurrently with course development to ensure that competencies drove content development.

*Process followed:*

- Defining the persona/career profile: Identified the target audience as “bioinformatics researchers.”
- Identifying outcomes: Determined specific goals and identified relevant skills and topics.
- Mapping to competencies: Mapped broad topic areas to ISCB competencies and Bloom's Taxonomy levels.
- Breaking down into KSAs: Broke down the competencies further into Knowledge, Skills, and Attitudes (KSAs).
- Matching assessments: Aligned course modules and topics with assessment mechanisms such as peer evaluation.
- Evaluation and iteration: Continuously evaluated assessments to identify gaps and make improvements.

*Unique step:* Using KSAs to drive content development.

*Major challenge:* Determining priority competencies and ensuring consistent mapping by multiple trainers.

**Design of a new university degree programme:** The Kwame Nkrumah University of Science and Technology in Ghana designed an MSc programme in BioData Analytics and Computational Genomics, guided by the ISCB Competency Framework to help design courses that would produce graduates who align with specific career profiles. This case study outlined the steps involved in programme development, from identifying target audiences to mapping competencies, Bloom’s Taxonomy levels, and attributes (KSAs).

*Process followed:*

- Defining programme aims: Established aims and objectives for the MSc programme.
- Identifying target audience: Determined the programme's target audience based on specific personas/career profiles.
- Mapping competencies: Mapped competencies to programme goals for each persona/career profile.
- Curriculum design: Designed courses and content to address the identified competencies.
- Mapping to Bloom’s Taxonomy: Mapped competencies to Bloom's Taxonomy.
- Review and improvement: Reviewed and improved content to ensure desired competency achievement.

*Unique Step:* Designing courses to produce graduates who align with specific career profiles.

*Major challenge:* Defining the depth of competency coverage and addressing biases.

The approach allowed for a tailored competency-based curriculum design and revealed gaps in the level of coverage of certain competencies currently covered by the program.

**Reviewing an existing training course:** H3ABioNet’s Introduction to Bioinformatics Training (IBT) Course, originally designed for molecular biologists, underwent retrospective mapping to the ISCB Competency Framework to assess alignment with proposed competencies for a particular persona/career profile. This mapping identified competencies well addressed by the course and areas requiring improvement. The process included mapping to Bloom’s Taxonomy and identifying KSAs.

*Process followed:*

- Persona/career profile identification: Identified the target career profile for the course.
- Competency mapping: Retrospectively mapped existing course modules to ISCB competencies.
- Bloom's Taxonomy levels determination: determined the Bloom's Taxonomy levels achieved by the course content.
- KSA Identification: Identified KSAs based on course content.
- Gap analysis: Identified competencies that were inadequately addressed or not addressed at all.

*Unique Step:* Retrospective mapping of an existing course to the ISCB Competency Framework to assess competency coverage and depth.

*Major challenge:* Mapping broad competencies and determining competency depth in an introductory course.

**Mapping existing training courses to design additional courses:** This use case involved mapping online and overseas bioinformatics courses offered by Wellcome Genome Campus Advanced Courses (ACSC) to the ISCB Competency Framework. The mapping aimed to identify competency coverage and gaps, informing the design of new courses. The study categorized courses into ISCB personas/career profiles and graded competency coverage. Feedback from bioinformatics experts and stakeholders was incorporated. The mapping helped identify gaps, refine course content, and inform training strategies.

*Process followed:*

- Categorizing target audience: Identified the target audience as equivalent to the ISCB persona “Discovery biologist.”
- Competency mapping: Mapped the syllabus of existing courses to ISCB competencies.
- Grading competency coverage: Graded competency elements as “Mostly covered,” “covered in part,” or “not covered.”
- Bloom’s Taxonomy levels and KSA determination: Analysed Learning Outcomes to confirm Bloom’s levels and identified KSAs.
- Feedback incorporation: Engaged in-house bioinformaticians and education teams for feedback.

*Unique step:* Grading competency elements and involving bioinformatics experts and stakeholders in feedback and gap analysis.

*Major challenges:* Content reviews required subject matter expertise.

**Reviewing a university degree programme:** UNSW Sydney’s bioinformatics degree programmes were designed with retrospective use of the ISCB Competency Framework. Programmes were built based on available courses, and their learning outcomes were mapped to competencies. This revealed competency gaps, which were subsequently addressed by revising dedicated bioinformatics subjects. The design considered not only the curriculum but also university and external body requirements. UNSW's approach emphasizes adaptability within a pre-existing academic environment.

*Process followed:*

- High-Level programme design: Designed programmes at a high level by selecting from available courses.
- Competency mapping: Mapped learning outcomes of courses to the ISCB Competency Framework, revealing competency gaps.
- Curriculum revision: Revised dedicated bioinformatics subjects to address competency gaps.
- Consideration of external requirements: Considered university and external body requirements, including degree accreditation.

*Unique step:* Adapting existing courses to competency-based goals and emphasizing adaptability within a pre-existing academic environment.

*Major challenges:* Often difficult to revise university programmes—and requires many approval steps.

In summary, each use case follows a process that involves identifying target audiences, mapping competencies, aligning content with Bloom’s Taxonomy, and conducting gap analyses. These processes collectively highlight the versatility of the ISCB Competency Framework in various educational contexts.

### 3.6 How can individuals use the framework?

**Figure 3.**
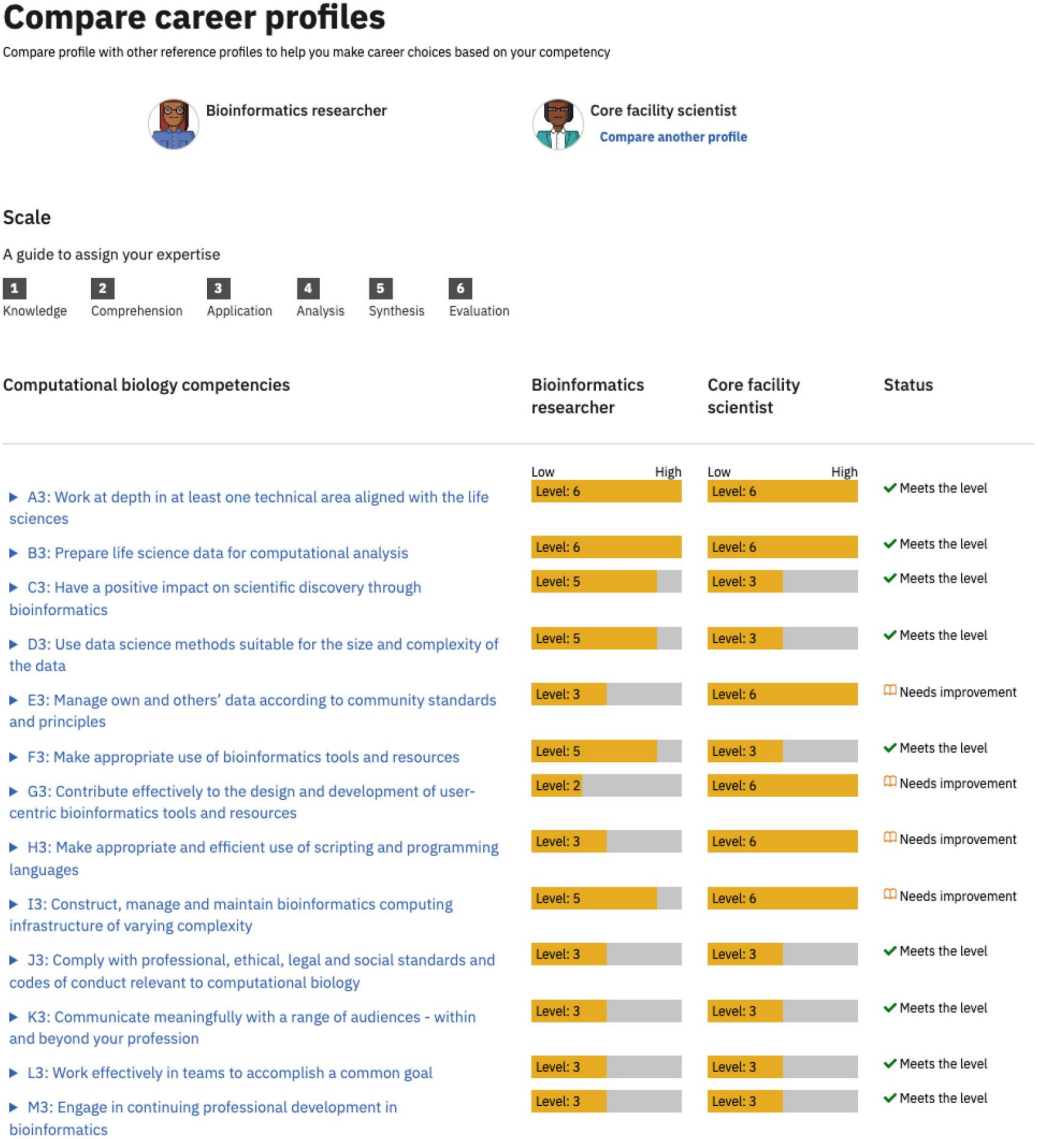
Comparison of two ISCB career profiles in the competency hub. A user can select two pre-published career profiles (here Bioinformatics researcher and core facility scientist) and compare the minimum Bloom’s level at which competency is required. A “status” column clarifies which competency areas the user needs to work on. Each competency in the hub is linked to courses that support users to develop their competency in that particular area (not shown).

Students and professionals in bioinformatics can use the ISCB competency framework to assess their own knowledge, skills, and attitudes against a standard elaborated by experts in the field. This will help them decide which competencies they need to develop further. In addition, the personas/career profiles provided in the ISCB competency framework can act as a reference for an individual to decide in which direction to develop in their career (Fig. 3). The career profiles that we created in the Competency Hub (https://competency.ebi.ac.uk/) include a short description of their background and activities associated with their current role, in addition to the level of competency required (e.g. see https://competency.ebi.ac.uk/framework/iscb/3.0/profile/view/8456/antonio). This is intended to provide sufficient information to support students and professionals to make informed decisions around whether a specific role is appropriate for them. Individuals can login and create a personal profile, capturing their own competency levels, and then compare this to the career profiles listed. They can also compare two or more of the published career profiles (Fig. 3).

Finally, the ISCB Competency Framework can inform students and professionals about the training or experience that they need to further develop their competency. This works especially well for courses that have been designed according to the framework and that state clearly which competencies or KSAs they help to build, as they relate to the same standard as the one that the individual is using for self-assessment.

### 3.7 Future plans for the ISCB competency framework

Version 3.0 of the ISCB Competency Framework is openly available through https://competency.ebi.ac.uk/framework/iscb/3.0/competencies. It is also listed as a resource on the the ISCB website (https://www.iscb.org/curriculum-guidelines-colleges-universities) and the GOBLET Trainer Resources portal (https://www.mygoblet.org/training-portal/trainer-resources/). Plans are being developed to publicize the framework and its application more widely through ISCB activities worldwide and through a broader communication strategy, to continue to engage the bioinformatics education community; this includes working with other scholarly societies and professional bodies whose members have a bioinformatics education and training requirement, including the clinical community. To continuously improve usability, sessions will be organized on specific use cases, such as mapping a short course or designing a new degree program. Users will be able to bring their course information to map to competencies with the support of the developers of the competency framework. More detailed work is also being done in the context of specific career profiles, with the goal of supporting career progression and better recognition of bioinformatics roles that sit outside of the classical researcher career pathway. For example, some members of the taskforce are working with BioInfoCore, the ISCB’s community for core facility managers, to define a competency-based career path for core facility professionals. When version 1 of the competency framework was developed (Welch *et al.* 2014), job postings were used to identify commonly requested competencies. We have not repeated this for versions 2 and 3, but for work related to specific career paths this could be a fruitful exercise—enabling us to respond to changing needs and incorporate them.

We have not yet sought feedback from individuals who have used, or are using, the framework to retrain or change roles in the workplace. As our labour market continues to evolve rapidly, especially in light of new disruptive technologies (e.g. the explosion in single cell and spatial omics, which is increasingly being used to understand development and pathology, or the rapid adoption of large language models and other AI methods to biodata annotation and modelling), it would be instructive to seek input from such use cases.

Computational biology is a fast-moving field that is becoming increasingly applicable to different professions. To remain useful and relevant, the ISCB Competency Framework must be updated regularly to cover emerging topics. Areas under consideration for the next version of the framework include mapping more transparently to the FAIR skills terminology, (https://terms4fairskills.github.io/), which aims to describe the competencies associated with making and keeping data FAIR. Work is also underway to extend the ISCB Competency Framework to more specific application areas, such as bioinformatics in clinical decision making or in agronomy. This will require the creation of new career profiles and the mapping of existing competencies to them, in consultation with a critical mass of individuals working in those application areas. We anticipate that this work will also reveal areas of competency that we have not, to date, considered.

We welcome new input into the ISCB competency framework; those wishing to participate in the development of version 4.0 are encouraged to join the ISCB Education COSI mailing list by emailing education_help@iscb.org and asking to be added.

## Supplementary Material

vbae166_Supplementary_Data

## Data Availability

The data underlying this article are available in the EMBL-EBI Competency Hub at https://competency.ebi.ac.uk/framework/iscb/3, and can be accessed with the unique identifiers ISCB_A3 to ISCB_M3.
Box 1: Ensuring relevance of the ISCB Competency Framework in a truly international context. Development of the ISCB Competency Framework has always given careful consideration to being representative of our field. Members of the ISCB Competency Framework working group: represent a diverse cross-section of bioinformatics professionals; incorporate expertise in different bioinformatics domains; include a wide range of geographical contexts; actively encourage application in different sectors; and trailblaze application to different types of learning interventions. Whilst working-group participants vary from one meeting to the next, the majority are affiliated with one or more of the following groups: Global Organisation for Bioinformatics Learning, Education and Training (GOBLET; Attwood *et al.* 2015) H3ABioNet (Pan African Bioinformatics Network; Mulder *et al.* 2016) ELIXIR (European research infrastructure for life sciences) EMBL-EBI (EMBL-European Bioinformatics Institute; Cantelli *et al.* 2022) SoIBio (Sociedad Iberoamericana de Bioinformática / Iberoamerican Society for Bioinformatics; De Las Rivas *et al.* 2019) CABANA (Capacity building for bioinformatics in Latin America) Wellcome Connecting Science Fogarty International-funded training programs in Africa
