# Supplementary material for: The ISCB competency framework v. 3: a revised and extended standard for bioinformatics education and training
Source: Bioinform Adv. 2024 Nov 18;4(1):vbae166. doi: 10.1093/bioadv/vbae166 (PMC11646570; doi:10.1093/bioadv/vbae166)
Supplement: vbae166_Supplementary_Data [file vbae166_supplementary_data.zip › Competencies_use_case_studies.pdf]

Case studies to support use of the ISCB Competency Framework for curriculum design and evaluation

Bruno Gaeta (1), Shaun Aron (2), Dusanka Nikolic (3), Rachel Berkson (3) and Alice Matimba (3), Cath Brooksbank*

1. School of Computer Science and Engineering, UNSW Sydney, Australia
2. Sydney Brenner Institute for Molecular Bioscience, University of the Witwatersrand, Johannesburg, South Africa
3. Wellcome Connecting Science, Wellcome Genome Campus, Hinxton, Cambridge, CB10 1SA
4. EMBL-European Bioinformatics Institute, Wellcome Genome Campus, Hinxton, Cambridge, CB10 1SD, UK

*To whom correspondence should be addressed

0. Summary

This document provides a few example case studies of applications of the ISCB competencies in various forms of course and curriculum design or evaluation. These brief examples are meant to be illustrative of the kinds of situations for which the competencies might apply and show how they were used in practice by members of the bioinformatics education community.

1. New training course case study: H3ABioNet's 16S rRNA Intermediate Bioinformatics Training (Int_BT)

(Shaun Aron)

H3ABioNet's 16S rRNA Intermediate Bioinformatics Training (Int_BT) provides a case study in how the competency framework can assist in the new course development process. Step 0 in the Nicholls cycle, defining a target audience, is aided by identifying a persona, in this case the "bioinformatics scientist" persona. Step 1, identifying outcomes, is dictated by the specific goal of this class, training in 16S rRNA analysis, as well as by some specific skills required for that domain – e.g., NGS technologies, R programming, and workflow

management – and broad topic areas, such as intro to R and intro to the Linux command line. The competency framework then allows one to map these broad topic areas into competencies and Bloom's Taxonomy levels, and from there break them down into KSAs that can be used to identify experiences (Step 2) and content (Step 3) at the level of course modules and topics. Those can then be matched to assessment mechanisms (Step 4), in this case, for example, peer evaluation and other course feedback mechanisms. The evaluation of these assessments (Step 5) then made it possible to identify gaps and iterate to a further round of improvement. For more information, see the detailed course map.

Experience with this class prompted a few observations. One is that it was significantly easier to determine and highlight competencies to be developed by the training when done during the planning phase than when done post-hoc. Content could then be developed with the goal/aim of uplifting participants to a particular level of competency in already identified areas. Doing so is much easier than first developing content and then needing to change it after gaps are highlighted through competency mapping. This approach of doing mapping concurrent with course development allows competencies to drive the development of the content and not the inverse. It ensures gaps are spotted before implementation and has proven valuable in the development of the course. On the other hand, the approach did face some challenges. Determining which competencies are the most important areas of focus and which are deemed minimum standards by the community can be difficult, as bias will always exist. It is particularly difficult with a course like this one that makes use of a variety of trainers to map competencies consistently. Furthermore, trainers often struggled to understand the personas and Bloom's Taxonomy, but all admitted that it became progressively easier over time.

Process followed:

- Step 1: Define the persona - for this example it is a bioinformatics scientist
- Step 2: Decide on the focus/main topic area - in this case it was the 16S microbiome
- Step 3: Based on step 2 – collect information on what the current required skills and competencies are in this area/domain. For Int_BT, NGS technologies, R programming, workflow management, etc. were some skills highlighted.
- Step 4: Define broad topic areas e.g.: intro to R and intro to Linux command line
- Step 5: Based on broad topic areas (defined by steps 2 and 3) determine what competencies should ideally be achieved via these modules/topics.
- Step 6: Map these competencies to bloom's taxonomy
- Step 7: Map KSAs specifically addressed by each module/topic
- Step 7: Summarize the competencies and KSAs that will be delivered by the course
- Step 8: Peer evaluation and feedback
- Step 9: Improvements in mapping and gaps identified

Key successes: This approach allows competencies to drive the development of the content and not the inverse. This ensures gaps are spotted before implementation and has proven valuable in the development of a course.

2. Design of a new university degree program: Kwame Nkrumah University of Science and Technology MSc Programme

(Shaun Aron)

The Kwame Nkrumah University of Science and Technology based in Kumasi, Ghana developed an MSc programme in BioData Analytics and Computational Genomics. The programme was designed to provide students with the analytical skills and expertise to obtain the bioinformatics competencies required in an academic setting as well as a core bioinformatics service environment. With this in mind, the program was developed using a previous version of the ISCB competency mapping framework. The process followed is outlined below. For more details, refer to the degree programming planning document, which includes a full curriculum map.

Process followed

1. Aims and objectives of the programme identified
2. Target audience determined (student background and eligibility very important)
3. Duration of programme determined
4. Determine personas: the programme was developed to produce graduates who can fit any of four personas, namely:
    i. Academic bioinformatics researcher
    ii. Core facility scientist
    iii. Bioinformatician in an academic or research infrastructure support
    iv. Bioinformatics software developer/ software engineer

   All courses were designed to meet the requisite competencies needed to produce the personas indicated above
5. Competencies to be developed by program determined for each persona
6. Courses/module and content designed and developed (syllabus and curriculum)
7. Competencies mapped to the personas above separately
8. Competencies then mapped to Bloom's taxonomy and KSA's
9. Content reviewed and improved where appropriate to deliver desired competency at a desired depth.

3. Reviewing an existing training course: H3ABioNet's Introduction to Bioinformatics Training (IBT) Course

(Shaun Aron)

H3ABioNet's Introduction to Bioinformatics (IBT) Course is a multiple-delivery-mode training approach developed to provide basic bioinformatics training to those individuals entering the

field of bioinformatics. The course has previously been designed for molecular biologists wanting to familiarise themselves with the field of bioinformatics. While the modules were designed to cover basic bioinformatic tools, the curriculum was not originally designed around any formal competency framework. The ISCB competencies framework was therefore used to retrospectively map competencies to the various modules of the course. In addition, competencies were then mapped to Bloom's Taxonomy and KSA's. The mapped competencies have subsequently been used to update and improve the content of each module. For more detail, refer to the full mapping.

While mapping IBT to the updated framework meant that it "lost" one competency (I), it "gained" competencies H and E2 (newly revised competencies). Mapping an existing course like IBT allowed identifying which competencies were well addressed by the course and at sufficient depth for the target audience, but also allowed a gap analysis to determine which competencies were poorly addressed or at insufficient depths (per Bloom's taxonomy). This will allow further improvement and refinement of the course content. Adding KSAs also allowed identifying key skills and knowledge areas not addressed by the course and highlighted these gaps.

The process involved some challenges, most notably that some competencies seemed too broad to be easily applied. It proved hard to differentiate between content that is just incidentally touched upon versus the content that is the real meat of the course. This was true for IBT especially since it is at an introductory level and many concepts may be touched on only superficially. Many who perform competency mapping found it inherently difficult to map to, or understand how to apply, the Bloom's taxonomy. Nonetheless, it was still highly valuable. It provided a snapshot of the depth at which a particular competency is addressed by the course and helped determine whether all competencies are addressed at significant depth or perhaps in too much depth for the level at which it was being delivered. It also allowed us to spot gaps in competencies covered for a particular persona at a particular level and thus allowed us to develop intermediate training to address some of these gaps.

Process followed

1. A persona was identified (see ISCB list of personas and suggested competencies) and relevant competencies were identified using a detailed course syllabus and curriculum.
2. Competencies were mapped to the old version of the framework using a spreadsheet. Each module was mapped individually as this allowed for the most accurate analysis of competencies covered.
3. Bloom's levels were then determined using the course's curriculum as a guide.
4. KSAs were then identified based on the content addressed and level of the course. In this case, it is an introductory course thus no KSAs dealing with implementation would be expected.
5. This process was then repeated using the updated framework and any changes to the competencies were recorded.
6. As unintended bias is often a major concern, multiple stakeholders were engaged for feedback and the mapping updated accordingly.

7. Gap analyses were performed to determine which competencies were poorly addressed or not addressed at all.

4. Mapping existing training courses to design additional courses: Wellcome Genome Campus Advanced Courses

(Dusanka Nikolic, Rachel Berkson and Alice Matimba)

Advanced Courses (ACSC) delivers a range of advanced professional training in research and applications of genomics to a wide range of audiences including biologists and healthcare professionals. The ACSC team mapped two programmes which have an international reach - Online course and Overseas courses. Three short online bioinformatics courses make up a programme covering aspects of bacterial genomics. These courses are run as MOOCs using the FutureLearn platform. The aim of the mapping was to assess what competencies are covered so far. The mapping results are helping us in the design of the fourth new online course in the bioinformatics programme by identifying the gaps against the bigger plan/strategy for WGC overall online courses (and/or wider) bioinformatics training. Vice versa , this can also inform our overall strategy through analysis of the bioinformatics training needs (for our target audience) using the ISCB core competencies framework.

As the ISCB framework represents a comprehensive set of competencies we did not expect our short courses to map into the entire set, rather they map into a smaller subset of competencies. The predominant target audience for our advanced online courses is equivalent to the ISCB persona *Discovery biologist/academic life science researchers*. We can assume background knowledge to be already on the highest Bloom's level for competencies on general biology and depth/specialisation in one of the biology fields (ISCB competencies A and B).

The syllabus covered in the three bioinformatics courses is mapped overall (the three short courses being treated as a single unit) into the detailed ISCB competencies to determine what content is covered. Grading is used to roughly say to what extent the particular element of the competency is covered. Elements were graded as *Mostly covered*, *covered in part* and *not covered*. The Learning Outcomes are checked for the Bloom's levels intended/achieved by the courses, based on the action verbs. All three courses have both formative and summative assessment and this can also be used to confirm the Bloom's levels for particular competency. The mapping was presented in a matrix using a spreadsheet, with rows representing detailed competencies, expanded with all the Knowledge, Skills and Attitudes relevant to each competency. Columns represent the rough level of competency and the Bloom's level of relevant Learning Outcomes. RAG method was used to visualise the coverage of the specific KSAs elements of a competency.

Link to mapping:
https://drive.google.com/drive/folders/1wU7IeF0YX_vpcK0AGDgHTXd97ctz-XSH?usp=sharing

The courses mapping was checked with in-house bioinformaticians and education team and feedback was implemented. We wanted to highlight the gaps in training we are providing through our online programme – those were identified and some gaps are being considered for coverage in the new bioinformatics course to be designed and developed for the FL platform by the team of internal and external experts bioinformaticians and ACSC education team. In particular, within the competency F: Bioinformatics tools and resources and their usage, a specific skill SF-8 on using the command line to solve typical bioinformatics tasks is considered for implementation in the new course. A similar approach is being used to map the bioinformatics competences covered in the ACSC overseas course programme. Again, we assumed the target audience would be biologists or life science researchers with expert knowledge in competencies A and B. Some competencies relating to e.g. seeking out appropriate training were inherent in the nature of the overseas courses.

5. Reviewing a university degree program: the Bachelor of Engineering (Bioinformatics Engineering), UNSW Sydney

(Bruno Gaeta)

UNSW Sydney offers two different undergraduate degrees in bioinformatics. One is a Bachelor of Engineering in Bioinformatics, the other is a Bachelor of Science major in bioinformatics. Both degrees share a number of courses but they have different target audiences and learning objectives, with the Engineering degree aiming to train developers and designers of bioinformatics applications, software and systems, and the Science degree aiming to train users of bioinformatics working towards discovery in biology (although there is significant overlap between the two).

The design of these programs is influenced by more than pure curriculum considerations. The programs must as much as possible make use of existing courses already offered by the university and must include the foundation courses required as prerequisites for these courses. The programs must also satisfy the rules imposed by university and external bodies such as the ones involved in degree accreditation.

The way the ISCB competencies were used were mostly retrospective. The programs were designed at a high level by selecting from available courses in the areas that should be covered (biology, computer science, mathematics, statistics, engineering). Then the learning outcomes of these courses were mapped to the ISCB competencies using the 0,1,2 mapping. This revealed “holes” in the program - competencies that were only partially addressed, or not addressed at all. The few courses over which the program designer had control - in this case the dedicated bioinformatics subjects, were then revised to address these holes. This involved for example adding some content on ethics in one bioinformatics course and adding some additional statistics and experimental design in another.
