# Supplementary material for: The ISCB competency framework v. 3: a revised and extended standard for bioinformatics education and training
Source: Bioinform Adv. 2024 Nov 18;4(1):vbae166. doi: 10.1093/bioadv/vbae166 (PMC11646570; doi:10.1093/bioadv/vbae166)
Supplement: vbae166_Supplementary_Data [file vbae166_supplementary_data.zip › Competency_guidelines.pdf]

Guidelines for developing and updating short courses and course programs using the ISCB competency framework

Russell Schwartz (1), Cath Brooksbank (2*), Bruno Gaeta (3), Nicola Mulder (4), Sarah Morgan (2), Venkata Satagopam (5), Verena Ras (4), Pandam Salifu (6), Sonika Tyagi (7), Gaston K. Mazandu (8), Shaun Aron (9), Marta Lloret Llinares (2) Judit Kumuthini (10), Michelle Brazas (11), Astrid Gall (2), Benjamin Moore (2), Alexandra Holinski (2)*

1. Department of Biological Sciences and Computational Biology Department, Carnegie Mellon University, Pittsburgh, Pennsylvania, United States of America
2. EMBL-European Bioinformatics Institute, Wellcome Genome Campus, Hinxton, Cambridge, CB10 1SD, UK
3. University of New South Wales, Sydney, Australia
4. Computational Biology Division, Department of Integrative Biomedical Sciences, IDM, Faculty of Health Sciences, University of Cape Town, South Africa
5. University of Luxembourg, 2, Avenue de l'Université, L-4365 Esch-sur-Alzette, Luxembourg
6. Kumasi Centre for Collaborative Research in Tropical Medicine, Kumasi, Ghana
7. Australian Genome Research Facility Ltd Melbourne, Monash University, Melbourne, Australia
8. Division of Human Genetics, Department of Pathology, University of Cape Town Faculty of Health Sciences, South Africa
9. Sydney Brenner Institute for Molecular Bioscience, University of the Witwatersrand, Johannesburg, South Africa
10. Centre for Proteomic and Genomic Research (CPGR), Cape Town, South, Africa
11. Ontario Institute for Cancer Research, Toronto, Canada

**To whom correspondence should be addressed:* russells@andrew.cmu.edu, cath@ebi.ac.uk

Contents

0. Summary **3**

1. Introduction **3**

1.1 What is a competency? 3

1.2. What is a competency framework? 4

2. The ISCB competency framework **5**

2.1 Where did the need to develop the ISCB competency framework come from? 5

2.2. Version 1: users, scientists and engineers 6

2.3. Version 2: Identifiable job roles and different levels of competency 7

2.4. Version 3: enriching the framework with knowledge, skills and attitudes 8

3. The course/curriculum design process **9**

3.1. Developing a full-length course as part of a degree program **10**

0. Identify the target audience 11

1. Identify outcomes 11

2. Select experiences 12

3. Identify content 12

4. Identify assessments 12

5. Check assumptions 13

6. Iterate 13

3.2. Considerations for developing or revising a full degree program **14**

3.3. Considerations for developing a program of short courses **15**

0: Who is your target audience? 15

1: What competencies do they need to acquire and at which level? 15

2-4: Putting it all together 16

5: Checking assumptions 17

6: Iterate and improve 17

3.4 Guidelines for mapping existing courses and degrees to the framework 17

0, 1, 2 mapping 18

Assessment weight mapping 19

4. Conclusion **21**

References **21**

Appendix **23**

A1. Related competency frameworks **23**

0. Summary

Competency frameworks have proved to be a powerful tool for curriculum development and assessment across many subject domains, and the field of computational biology is no exception. Efforts from the ISCB to develop and successively refine a set of competencies for bioinformatics education and various associated mapping tools have provided a framework for bringing competency-based design principles broadly to education and training of a wide range of professionals in need of some level of mastery of the principles and practice of computational biology. This document seeks to provide some basic guidance for education and training professionals in the field in how to use this framework effectively. It includes a basic background on competency-based education and the history of the ISCB competency framework specifically, leading up to the Version 3 framework considered here. It then follows with some basic principles of applying competency-based education and an illustration of how they apply to different tasks in curriculum development. Appendices and various linked documents provide further elaboration and helpful guidance on the ISCB competencies specifically and some ways in which versions of them have been used already to develop diverse forms of bioinformatics education and training experience. Our target readerships are trainers and educators working in computational biology or more broadly in the molecular life sciences, medicine, and other disciplines that use biomolecular data, including those working in academia, industry and the public sector.

1. Introduction

1.1 What is a competency?

By definition, a competency is an observable ability of any professional, integrating multiple components such as knowledge, skills, values and attitudes. It is observable, so its acquisition can be validated objectively. The notion of competency provides a shared 'currency' applicable to learning of all types and at all career stages. Examples of uses of competencies include:

1. Competency allows individual professionals to assess themselves against a professional standard, and seek appropriate continuing professional development and/or develop their own careers.
2. Professional bodies can use competency to define **competency frameworks** for different roles or professions. These can form the basis of recognised professional standards, especially in regulated professions, for example healthcare and engineering.
3. Learning professionals can use competencies to develop training or other learning interventions, by asking what competencies their learners need to gain.
4. Learning professionals can ascertain whether training delivered has enabled learners to gain a competency or a set of competencies, by assessing whether

knowledge has been transferred, skills have been developed, and/or behaviours/attitudes have changed as a result of the training.
5. Competency can be used to determine whether curricula or training materials meet professional standards, identify course providers who can deliver training of the required quality, and support trainers to meet or exceed the required standards.
6. Competency can be used by employers to assess skills requirements, write job descriptions, determine whether candidates are appropriate for roles, and support employees to develop further in their roles.

Competency-based practices have become ubiquitous in modern education and training and there are now numerous resources to which the interested reader might refer for more information and case studies of their use in different fields and contexts. See, for example, (Burke 2005) for broader coverage of competencies and their uses and (Welch et al. 2014; Mulder et al. 2018; Wilson Sayres et al. 2018; Davies, Mueller, and Moulton 2020) for some examples of competency frameworks in bioinformatics and related fields.

1.2. What is a competency framework?

A competency framework (or profile) defines the competencies required to fulfil a particular role or activity. Competency frameworks are typically defined by professional bodies or learned societies in collaboration with employers and learning professionals for a specific purpose. For an extended notable example, one might refer to the U.K. National Occupational Standards (https://www.ukstandards.org.uk/). See Appendix A1 for some representative examples of competency frameworks in bioinformatics and beyond.

Although there is no accepted common or universal format for a competency framework, there are many common elements. Typical frameworks summarise a core set of competencies required by professionals working in a defined field. Each competency may have a detailed definition, and groups of competencies may be organised into themes or areas. Some frameworks specify which competencies are especially relevant for professionals in different roles or at different career stages, and some incorporate different levels of competency requirements (Table 1).

While competency frameworks can be a valuable tool for education and training, applying competency frameworks to curriculum or course development is not straightforward and requires caution. Many competency frameworks exist, created and developed for different purposes. Because there is no universally accepted format for doing so, it can be difficult to compare frameworks and choose an appropriate one. Further, competency frameworks can be time-consuming and cumbersome to develop and keep up to date. They are only useful if they undergo regular review by experienced professionals working in the roles covered by the framework. As a result, those seeking to develop or employ a competency framework can expect to require guidance from those who have experience or skills in the field.

Table 1: *Example of a generic template for a competency framework.*

Theme	Competency	Requirements for role 1, entry level	Requirements for role (1+n), senior level
Theme 1	Competency 1a short description<br>• Knowledge<br>• Skills<br>• Attitudes	Select from, e.g. …<br>0 - Not required<br>1 - Awareness<br>2 - Working knowledge<br>3 - Specialist expertise	Select from, e.g. …<br>0 - Not required<br>1 - Awareness<br>2 - Working knowledge<br>3 - Specialist expertise
	Competency 1b short description<br>• Knowledge<br>• Skills<br>• Attitudes	Select from, e.g. …<br>0 - Not required<br>1 - Awareness<br>2 - Working knowledge<br>3 - Specialist expertise	Select from, e.g. …<br>0 - Not required<br>1 - Awareness<br>2 - Working knowledge<br>3 - Specialist expertise
	Competency 1c short description<br>• Knowledge<br>• Skills<br>• Attitudes	Select from, e.g. …<br>0 - Not required<br>1 - Awareness<br>2 - Working knowledge<br>3 - Specialist expertise	Select from, e.g. …<br>0 - Not required<br>1 - Awareness<br>2 - Working knowledge<br>3 - Specialist expertise
Theme 2	Competency 2a short description<br>• Knowledge<br>• Skills<br>• Attitudes	Select from, e.g. …<br>0 - Not required<br>1 - Awareness<br>2 - Working knowledge<br>3 - Specialist expertise	Select from, e.g. …<br>0 - Not required<br>1 - Awareness<br>2 - Working knowledge<br>3 - Specialist expertise
	Competency 2b short description<br>• Knowledge<br>• Skills<br>• Attitudes	Select from, e.g. …<br>0 - Not required<br>1 - Awareness<br>2 - Working knowledge<br>3 - Specialist expertise	Select from, e.g. …<br>0 - Not required<br>1 - Awareness<br>2 - Working knowledge<br>3 - Specialist expertise

2. The ISCB competency framework

The ISCB competency framework defines the core competencies required by professionals working in fields related to computational biology. It provides a minimum information standard defining the competencies required, and the levels they're required at, for a range of roles that require bioinformatics expertise, and it provides a tool to support bioinformatics educators to develop courses and curricula that meet the needs of employers. Note that we are using here the terms "computational biology" and "bioinformatics" interchangeably owing to the inconsistent use of both in the relevant literature. In its latest version (version 3.0 as of this writing), the ISCB competency framework specifies 13 competencies (labelled A to M), capturing the scientific, technical and professional competencies required by individuals working in different roles that have a significant requirement for computational biology.

2.1 Where did the need to develop the ISCB competency framework come from?

Training programs specifically in bioinformatics began to appear sporadically in the late 1980s and throughout the 1990s, although generally guided by the particular

experiences of self-taught practitioners. In 1998, Russ Altmann initially proposed a common curriculum for bioinformatics degrees. In the ensuing decade, numerous degree programs were developed — primarily at master's level, as well as at undergraduate and doctoral levels — to address the enormous demand for trained bioinformaticians. The Education Committee of the International Society for Computational Biology (ISCB) took up the task of trying to understand this landscape and provide guidance to those seeking to navigate it. Fast forward to 2011: The ISCB's Education Committee set up a task force to address the frustrations of bioinformatics employers, who were finding it extremely challenging to hire graduate-level employees with the 'right' skill set. An initial survey of educators in the field produced some very rough guidelines to current practice but largely highlighted a need for more information (Welch, Schwartz, and Lewitter 2012). The task force then conducted a broader survey of job descriptions, existing bioinformatics degree programs, and core facilities managers to get a feel for the skills required by a 'typical' bioinformatician and the range of practice among educators, and to work out where educational gaps were between them. At the time, the goal of the task force was to build a 'standard curriculum' that would fully address the needs of employers. However, it proved extremely challenging from the information available on course websites to identify common themes and topics. Course titles were often non-descriptive (e.g., 'Bioinformatics 101') and provided little or no information on learning objectives or outcomes. Furthermore, there was little apparent overlap between different training programs, or between training programs and the competencies that employers seemingly expected of program graduates (Welch et al. 2014). This issue was exacerbated by the fact that bioinformatics degrees and programs were often built in large part from pre-existing course offerings and needed to share their newly created courses among multiple training programs serving different cohorts. These difficulties prompted a great deal of debate on what guidance, if any, those working in bioinformatics education could provide to others seeking to enter the field, employ practitioners, or develop their own training programs.

2.2. Version 1: users, scientists and engineers

At the 2012 Intelligent Systems for Molecular Biology (ISMB) Conference (Long Beach, CA, USA), members of the ISCB education community debated whether it was necessary to look at course content at all. Some of our community had been working on defining competency frameworks in contexts beyond bioinformatics. These were gaining ground in the pharmaceutical industry and in clinical research and were already well established in some regulated professions such as healthcare and engineering. We wondered whether defining outcomes or required competencies, regardless of the route to gaining them, might unblock us. This is where the idea for a computational biology competency framework took root. Over the next year, the task force worked on the first draft of this framework, which was published in 2014 (Welch et al. 2014). We recognised at a very early stage that there were several different types of computational biologists. To develop version 1 of the framework, we created 'proto-personas' representing the different types of professionals that we had in mind. We labelled these initial personas the bioinformatics user, the bioinformatics scientist and the bioinformatics engineer.

Nonetheless, we recognized that this was a first pass at defining competencies that would need broader community input before it could become a widely adopted standard.

2.3. Version 2: Identifiable job roles and different levels of competency

Over the next few years, we sought input on the framework at workshops all over the world run under the auspices of the ISCB and the Global Organisation for Bioinformatics Learning, Education, & Training (GOBLET). These workshops asked for feedback on the competencies themselves and recruited trailblazers from the bioinformatics community to develop (or redevelop) education and training using the framework as a basis. The most consistent feedback that we got during this extensive consultation period was: (1) participants did not understand what the terms 'bioinformatics user/scientist/engineer' meant, and (2) participants needed guidance on how to use the framework to develop their own courses and degree programs.

To address the first issue, we refined the framework to include more identifiable job roles. The 'user' role was expanded to cover a broader set of job roles in which one might need to make use of bioinformatics tools, specifying separate roles for a physician, lab technician, ethicist and biocurator as personas for which one might require training in bioinformatics. The 'scientist' role was expanded to cover several roles in which one might need to apply bioinformatics for scientific discovery, including discovery biologist, molecular life science researcher, molecular life science educator, bioinformatics researcher, and core facility scientist. The 'engineer' role was expanded to cover different job roles developing bioinformatics tools and infrastructure in academic, industry, or other environments, including a bioinformatician supporting a lab, department, or research infrastructure and a software developer/engineer in a bioinformatics role. While even these broader roles could not be exhaustive, they provided a more comprehensive framework of the range of possible training needs within the field and models for how one might apply them to define other roles in the future.

The second issue arising from the surveys highlighted a challenge apparent since the ISCB's first survey efforts: the difficulty in finding a level of abstraction for specifying competencies that was broad enough to cover the wide range of practice in the field but narrow enough to be meaningful. In particular, it was necessary to solve the problem of how to specify the differences between diverse roles that might draw on the same competencies but with vastly different training needs in each. These roles were sufficiently varied that we felt it necessary to specify different levels of competency required for each role. After much discussion and broad consultation, including several more workshops at ISCB-affiliated meetings, we agreed to use Bloom's taxonomy (Bloom et al. 2001) to specify the level of each competency required for each role. A minimum of two task-force members independently specified these levels and we then cross-checked our assignments and amended them to reach a consensus. This work led to the publication of Version 2 of the framework in 2018 (Mulder et al. 2018).

2.4. Version 3: enriching the framework with knowledge, skills and attitudes

By the time the ISCB education community met at ISMB in 2018, several among us had used the framework to develop courses or curricula, some of us had mapped our existing programs to competencies, and some of us had gained experience of developing competency frameworks in different contexts. One of our chief concerns at this point, again backed up through community consultation, was that some of the competency definitions could be interpreted in numerous ways. It remained a challenge to find the right level of abstraction for competencies to be both tractable and useful, with a frequent concern among workshop participants being that the competencies were too coarse-grained to provide useful guidance. We considered the fact that others (e.g. RItrain.eu, corbel-project.eu, ESA) had overcome this issue by considering each competency as a combination of knowledge, skills and attitudes (KSAs), and agreed to work towards defining these attributes of the ISCB competencies. Our reasoning was that this would make it easier to define learning content and learning outcomes related to each competency, and would therefore support the bioinformatics training community's use of the framework. We were also conscious that it would help individuals to recognise whether or not they need to develop specific competencies.

The effort to rethink the competencies in terms of KSAs was carried forward through the Global Bioinformatics Education Summit, beginning in 2019 in Cape Town, South Africa and continued with a virtual meeting hosted in 2020 by the EMBL European Bioinformatics Institute in Hinxton, UK. These meetings prompted a great deal of effort to redefine each competency in terms of KSAs while stimulating a reorganisation of the competencies. Our discussions also led to a debate about the right level of resolution at which to apply these more detailed competency specifications to different personas. Ultimately, we decided to seek a consensus set of KSAs for each competency that could describe its use across all roles, while continuing to rely on Bloom's taxonomy to specify differences in levels of mastery of that competency between roles.

The final product of this effort, version 3.0 of the ISCB competency framework, includes an updated list of competencies and the knowledge, skills, and attitudes (KSAs) that define them and a mapping to a series of roles through Bloom's taxonomy. These guidelines have been created to support bioinformatics educators and trainers to make use of version 3.0.

3. The course/curriculum design process

In this section, we examine how the competency framework can be applied to a variety of tasks for designing, revising, or assessing short courses and degree programs. We consider several more specific case studies in the subsections below. However, there

are some high-level themes that apply across many potential uses. We therefore open with a brief description of the processes one might apply in using the competencies either to design a new training program or to help us to understand an existing one.

Figures 1 and 2 summarise the general processes illustrated by each of these applications. Figure 1 provides a general flowchart for applying the competency framework to design a new instructional program, which might be a short course or a full degree program. Figure 2 provides a similar process for mapping competencies to an existing program, for example to assess a program or suggest revisions. More details on each of these processes are provided in the subsequent sections.

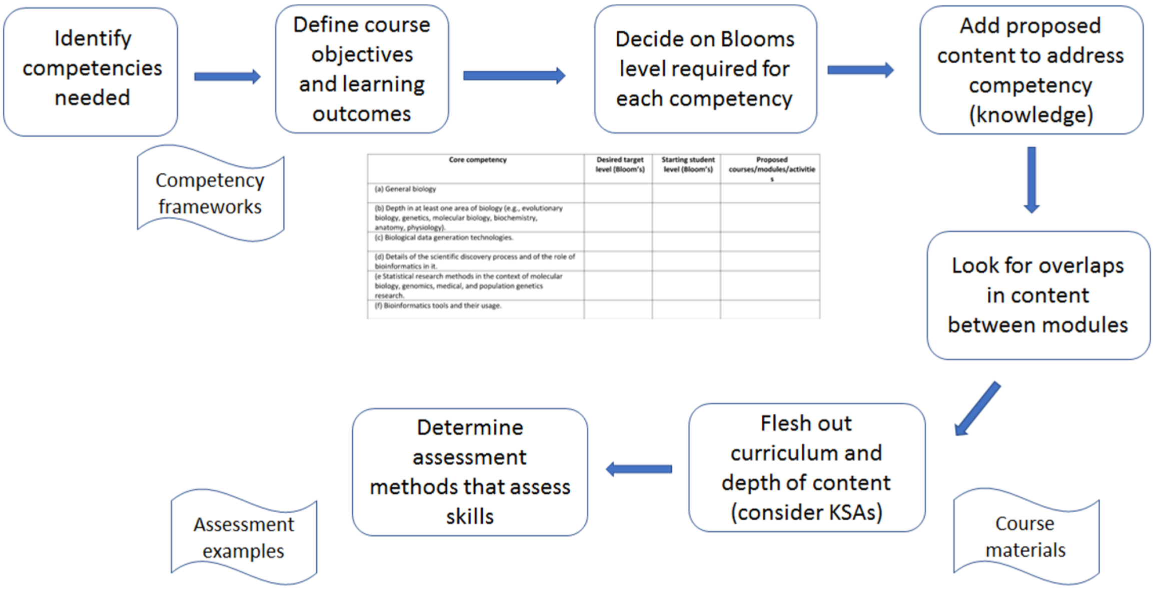

Figure 1. *Overview of the process for designing a new course using the ISCB competency framework*

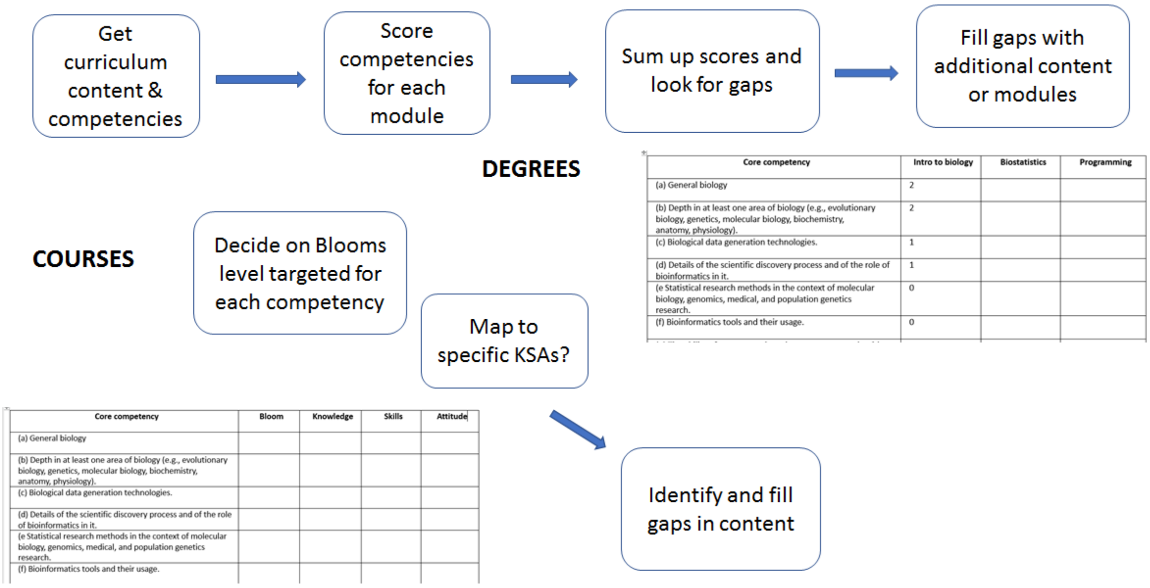

Figure 2. *Mapping competencies to an existing course.*

3.1. Developing a full-length course as part of a degree program

We begin by describing the development of a new full-length course using competencies. The process has significant overlap with that for designing a series of short courses described below, although there are also important differences created by the larger scope and time investment, for both educators and students, in a full-length course or degree program. It provides a useful starting point for describing some principles that will apply as well to other curriculum design needs.

When designing a new course, we suggest following a version of the Nicholls cycle (Nicholls 2002) (Figure 3):

(0) Identify the target audience
(1) Identify the outcomes we expect for that cohort from our course
(2) Identify experiences we think appropriate to achieve those outcomes
(3) Identify content to provide the appropriate experiences
(4) Identify assessments mapped to the intended outcomes
(5) Evaluate the results of the assessments.

The Nicholls cycle is essentially an elaborate variant of the questions we ask when developing a program of short courses. Our goal is to establish a process of continuing evidence-based improvement known as formative assessment. These steps can then feed back into a new iteration of course refinement. We assume here that you are using the ISCB competency framework; however, the steps are largely generic to course design and could be applied to other frameworks or training needs.

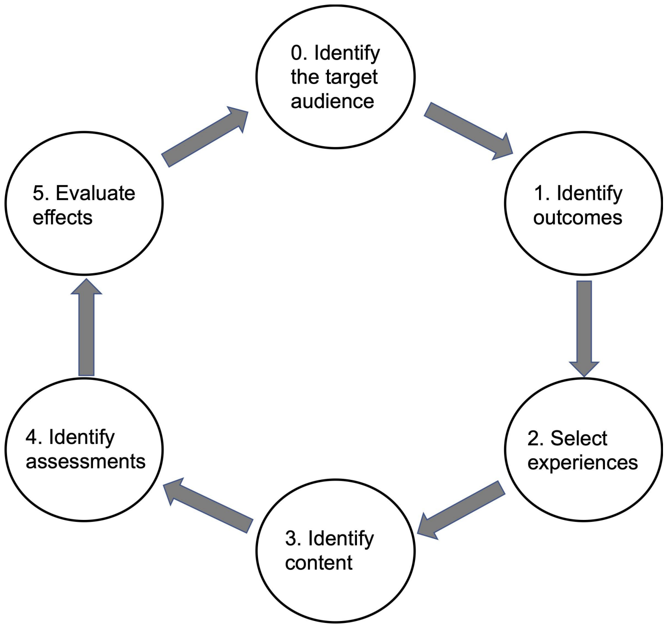

Figure 3: *Illustration of the Nicholls cycle for formative assessment (Nicholls 2002).*

0. Identify the target audience

We begin by identifying our target audience. In the simplest case, this might be a well-defined student cohort that has undertaken a standardized set of educational experiences before they arrive at our course. More often in an interdisciplinary field like bioinformatics, we have heterogeneity in our student population, with students entering with different backgrounds and varied prior training. In either case, we want to begin by identifying the common knowledge or experiences we can assume of that population. The more heterogeneous the population, the more challenging the process. A course designer might need to limit this heterogeneity via course prerequisites (specific foundation classes or other evidence of competencies required to take our new course), entry requirements (e.g., pre-testing), or mechanisms for remediation. Alternatively, we might make specific statements to entering students of the expected incoming competencies and resources by which they might check for themselves whether they have the necessary skills or need to acquire them before starting the course. Whatever prerequisites we identify, we will want to specify the minimum prerequisites for our program, also mapped to the competencies and KSAs we will expect of our incoming class.

1. Identify outcomes

We next need to identify our outputs: the competencies that we expect our students to possess when they leave our class. This, too, might be constrained to match the prerequisites of other courses later in the curriculum or to cover diverse future needs. In the simplest case, our course fits into a single full curriculum and we have already gone through the exercise of identifying the competencies and KSAs to be provided by each course in that curriculum (see Sec. 3.4: 'Guidelines for mapping existing courses and

degrees to the framework', below). In that case, we know the minimum competencies our course must provide to give the students the prerequisites for the next stages in the curriculum. More often, a course serves diverse needs and we need to do our best to find the union of the competencies or their KSAs required across the populations that it serves.

2. Select experiences

When we have identified the KSAs we must impart, we can then identify the kinds of experiences best suited to those specific KSAs. There are numerous options and a great deal of research on which learning experiences are more effective (e.g., Magana et al. 2014). Lectures are a natural and versatile choice for imparting knowledge, although often a deprecated one. More interactive variants, such as small break-out recitations or flipped classrooms in which we combine recorded lecture materials with in-class discussion, may prove more effective. Assigned readings, online instruction, and automated interactive systems may all be options. In bioinformatics contexts, interactive computer laboratory sessions are often appropriate. In each case, we must consider our resources — e.g., the physical infrastructure, tools/technologies and personnel to which we have access — as well as constraints under which we might need to operate with respect to delivery mode — e.g., in-person, virtual, or blended. Self-directed or peer learning, through individual or group projects and homework assignments, may be more valuable for developing skills and attitudes. Depending on the KSAs to be imparted, experiences involving teamwork, writing, or presentation may themselves be important tools for learning. Whatever our professional judgment on how to do this, it is advisable to revisit our experiences later through a process of formative assessment.

3. Identify content

Once we have identified the kinds of experiences we want to provide, we will need to identify the content to deliver through those experiences. The KSAs should provide a rough guide to the needed content, but we must rely on our own professional judgment and research to elaborate these into detailed content. We may have a good starting point, such as an earlier version of the class we are adapting, a good textbook on the subject matter, or a review article for more current content. For new or rapidly evolving topics, we may have to go to the primary scientific literature or rely on our own professional experience for content. We can also increasingly take advantage of the content available through third-party sources. It is necessary to follow good scholarly practice (e.g., use content to which we have legitimate access and cite our sources appropriately) but such materials are now often available in open or ideally FAIR (Wilkinson et al. 2016) formats and a number of prominent efforts are collecting large repositories of such materials (see, for example, the GOBLET Trainer Portal and FAIRsharing.org).

4. Identify assessments

The next step in good andragogical practice is to identify our assessments: how will we evaluate whether the intended competencies have been imparted successfully? As with

experiences, we have many options, both traditional and modern, and need to rely on professional judgment and the literature on effective mechanisms of assessment (e.g., Campbell and Nehm 2013). Traditional assessments include quizzes and written exams, homework/coursework assignments, course projects, grading hands-on experience in computer or other laboratory sessions, and written or oral presentations. In more modern variants, such assessments may be wholly or partly automated — such as through computerised multiple-choice questions — and such automated assessments have become an increasing concern for scalable technology-assisted learning. Assessments may also include seeking student feedback (course evaluation), having colleagues provide feedback on our class sessions or teaching materials (peer assessment), or evaluating other products of student achievement (material artefacts of course projects, for example). Assessments may be post-hoc, such as evaluating student performance in courses or research experiences following our own or tracking long-term performance on standardized tests or job placements, for example. For best practices, assessments should be mapped specifically to the KSAs we seek to impart and to the intended learning outcomes for the course. Assessment instruments often serve a dual purpose of evaluating the performance of specific students, e.g., for grading or planning interventions, and also facilitating formative assessment, in which we improve our course on the basis of the assessments. For this latter purpose, we often also will want to consider pre-course assessments or paired pre- and post-course assessments to determine the value added by our course as a whole or by specific modules or experiences.

5. Check assumptions

While it may seem obvious to evaluate the assessments of which we make use, it is important enough to be worth highlighting as a separate step. We want to know whether our students are reaching the intended level of mastery for each competency. If they are not, we need to identify for which competencies or KSAs the course is falling short and which experiences or course materials are not meeting our goals in that regard. This practice should involve revisiting our course competencies, KSAs, and the level of mastery expected in each on entry and exit from our course and identifying specifically where our assumptions or outcomes were as expected. This is not typically an either/or outcome. We succeed to different degrees and with different students and there is always room for improvement. As we move into the cycle of formative assessment, these evaluations will allow us to identify whether changes lead to improvement from one iteration of a course to the next or even on the fly during a single offering.

6. Iterate

Our assessments prepare us to return to step 0 through the process of formative assessment. We may find that our course fails to impart needed KSAs, in which case we can identify the inadequate experiences and propose alternatives. We may find that our assessments failed to give useful, actionable data and themselves need revision. Change may alternatively be imposed from without. For example, we may find that our students lack the input training we were led to expect, or that they come in better

prepared than expected, and we may need to adjust our planned experiences accordingly. Similarly, our audience may change, their needs may evolve, or those of the curriculum as a whole for our class may evolve. If our starting cohort has changing needs for competencies, we will probably need to change our outcomes, experiences, content, and assessments accordingly. In all cases, it is important to work through the full Nicholls process even in a course revision.

3.2. Considerations for developing or revising a full degree program

Degree program design or revision involves satisfying multiple sets of constraints. The competency framework provides targets in terms of overall learning outcomes but many other factors need to be considered, which may influence the content and structure of the program. Among the most important to consider are what other courses are available in your institution, the prerequisites necessary for students to enrol in the courses you identified, as well as institutional/national regulations and professional standards. This is not intended to be an exhaustive list, and others may be of similar importance in particular contexts.

Integrating one course with a full degree program presents challenges in large part because we will often have no control over material taught by colleagues or other departments and will be able to make changes only in a limited number of courses. Basing a curriculum on a well-defined competency framework and consistent use of objectives and learning outcomes program-wide may help to minimize the variability of materials taught outside our direct control.

Working with professional and national standards can present similar challenges as we may have little or no ability to influence them or to encourage them to work within a common competency framework. When considering other professional and national standards, it can therefore be useful to map them to the ISCB competency framework (or vice versa), by looking at overlaps between KSAs. This can simplify the mapping of multiple frameworks to the program and in turn provide us with a clearer basis for developing a program that meets the requirements of the standards in question.

When revising an existing degree program, we must make similar considerations even if our expectation is largely to continue to use pre-developed course materials and standards. Working with the competencies can help us to identify areas that are missing or looking too thin to meet the needs of our target audience. They can also identify content that is redundant and can be removed to make room for more useful content.

3.3. Considerations for developing a program of short courses

We next consider how the competencies can be used in the context of developing short courses, which we define as training experiences on a focused topic over a limited period of time. A typical short course might range from half a day to a week or two, covering a specific topic related to a subarea of computational biology. You might wish to design a single short course in isolation or a program of short courses providing training opportunities across a wider range of topics, as we consider here.

When developing a program of short courses there are several issues to consider before we can start applying or mapping competencies to our proposed program. Here, we enumerate some of the key considerations:

0: Who is your target audience?

Defining our target audience and identifying their goals in attending the training (akin to step 0 of the Nicholls cycle, above) will enable us to focus on the most appropriate set of competencies for that audience. Some questions we might ask about our audience include:

- Do they have a specific job/career/role?
- Does this role map to one or more of the currently defined ISCB roles?
- Are there specific tasks or responsibilities within that role for which they want to be prepared?
- What is their current career stage?
- What relevant prior training have they had?

1: What competencies do they need to acquire and at which level?

If our audience maps to one of the ISCB roles, we can then zoom in on the competencies that are required of professionals in this role, and the Bloom's taxonomy levels to which they are required according to the ISCB mappings. There may be other frameworks in which we want to consider this question (see Appendix A1), but the ISCB mappings should provide a good starting point for a variety of recognized roles for which bioinformatics training is required. This will help us to craft (or amend) appropriate learning outcomes and prioritise a list of topics to cover in our course program, and is roughly equivalent to Nicholls step 1.

Given our target audience and a good idea of the competencies they need to acquire, we can next ask if there is a specific level of training they require in each of those competencies. If we have already identified which ISCB competencies are relevant to the audience, we can then use the framework to guide us in two further ways:

1. Which Bloom's level does the training need to reach for each competency? For example, we might determine that the intention for our program is that our trainees move from knowledge of an area to the ability to apply that knowledge.

The ISCB framework defines the expected Bloom's levels for the professional roles it considers. We might take these definitions as suggested or use them as a starting point for defining needs for comparable roles not yet considered. In a short course, increasing the Bloom's level of a competency by more than one tier is probably unrealistic.

2. Are there specific areas of knowledge, skills or attitudes (KSAs) that are essential to our target audience? Version 3.0 of the ISCB Competency Framework provides KSAs that the community believes define a particular competency. In designing material for a short course, we might use these KSAs to suggest endpoints of our training and possibly content, learning experiences, and assessments aligned with those endpoints.

One additional thing to consider here is whether our target audience (or a significant subset of it) needs to demonstrate competence within another professional framework or to another specified standard. If this is the case, we might wish to compare the framework that our audience uses with the ISCB framework and create a simple mapping. For example, some of our authors (Larcombe, Brooksbank and Morgan) have previously mapped from the UK National Occupation Standard (NOS) and the UK Level 7 apprenticeship standard to version 2 of the ISCB competency framework (see this mapping table for more information). If we have already mapped our program to one of these standards, we will then be able to see which competencies are not covered, and this will help us to design a program that fulfils the competency requirements.

2-4: Putting it all together

Considering our answers to the questions above will help us to decide which competencies to focus on within our program. It is not expected that a program of short courses should deliver training that covers the whole ISCB competency framework; a subset of competencies delivered across a range of levels (for example, knowledge to application, or application to evaluation) may be more appropriate within our course delivery setting. If we are starting with an audience that already has a strong biological background, we might be able to assume that our audience already has considerable knowledge regarding competency A (General Biology); if they come from a computational background, we might be able to assume competency G, which includes topics in computer science systems and theory; if we have a mixed audience, we might assume neither of these things; while for an audience of experienced computational biologists we might be able to assume both. These considerations are not trivial, particularly if we cannot assume that our audience's prior preparation has been organized according to the same competency framework, and we may need to do some of that mapping ourselves or design our own pre-course assessments to accomplish this most effectively. Nonetheless, these types of factors can help us to consider not only which competency areas to focus on, but also how to word our course prerequisites and what we need to ask our target audience before a course begins.

Having decided which competencies are required by our audience, and to which level they need them, we can then define the experiences, content, topics and (if appropriate)

evaluation methods that will allow our learners to develop and demonstrate these competencies – roughly equivalent to steps 2–4 in the Nicholls cycle (see Sec. 3.1. 'Developing a full-length course as part of a degree program'). Each course within the program, once developed, should also then be mapped back to the key competencies identified to determine the extent to which they provide learners with the opportunity to develop a competency or set of competencies (see Sec. 3.4. 'Guidelines for mapping existing courses and degrees to the framework').

5: Checking assumptions

Demonstrating competency gained in a short course can be more difficult than demonstrating it for a degree program, as short courses seldom include a formal summative assessment. Short course providers are, however, encouraged to incorporate some form of formative assessment or evaluation, which will allow both learners and trainers to determine whether the required competency has been acquired. If learners are progressing towards higher levels of competency — application, synthesis or evaluation — it might not be possible to determine whether competency has been acquired during the time frame of the course. In all likelihood, learners will need to return to the workplace, apply what they have learned in their own setting, and adapt it to their own professional needs. Long-term surveys that ask trainees, 6 months or more after completion of a course, to evaluate their own level of competency can be useful here.

6: Iterating and improving

Proactively taking note of learner and trainer feedback, and revising future iterations of a course to address any challenges encountered, is just as important for short courses as it is for full-length ones. It is good practice to have a 'washup' meeting after each iteration of a short course. As well as considering any feedback from the trainees and specific challenges met by trainers (such as inadequate time to complete a learning experience, or a mixed target audience with varied learning needs). Such a meeting is a good opportunity to consider the latest version of the framework. Have there been changes to the framework that you might want to incorporate into future iterations of a course? Or have the needs of your trainees shaped a course in such a way that the framework is missing key KSAs? If the latter is the case, we warmly encourage you to contact the ISCB Education Committee via the corresponding authors of these guidelines; we are committed to the continuous improvement of the ISCB Competency Framework and this type of input is invaluable to us.

3.4 Guidelines for mapping existing courses and degrees to the framework

There are many reasons why we might seek to map an existing course or degree program to the ISCB competency framework. Doing so can be a useful evaluation tool to satisfy us that our program meets its intended learning objectives or to identify potential areas of improvement. Mapping might be part of a formal evaluation process, such as an accreditation review. It might also be preparatory to considering development or integration with other programs that may share coursework with our own. A single course or course module might serve needs for multiple different personas or even quite different training programs. For example, one module in a bioinformatics training program might also be relevant to a computer science program while a different module might be relevant to life sciences training outside the context of bioinformatics. Finally, conducting a mapping can be useful for better understanding the professional roles that a course or degree program serves or finding how to adjust the learning objectives to better serve an intended role.

The key process involves mapping the learning objectives and/or outcomes of the course to competencies and associated KSAs by reviewing these objectives or outcomes and identifying where they overlap or where there are gaps between them. The resulting catalogue of overlaps and gaps provides a guide for identifying which competencies are developed by participation the course or degree program, and which are not. These, in turn, help us to identify necessary revisions in the program to fill any identified gaps.

If the course includes student assessment, it is also useful to examine the assessment mechanisms. Examples of such mechanisms are discussed above in Sec. 3.1.4. In a well-designed course, these items should assess the learning outcomes, with some assessments also mapping to competencies incidentally. For example, an assessment that involves giving a presentation or writing a report implies that the course also maps to the communication competency (competency K3), while a group project might map to the teamwork competency (competency L3). Using the assessment items instead of, or in addition to, working directly with the learning outcomes can provide a more quantitative and consistent mapping based on the proportion of total marks mapping to a given competency.

A degree program consisting of multiple courses can be mapped to the framework by collating the competencies to which each of its component courses map. In its simplest form, this mapping can be true/false (a competency maps to the program if it maps to one or more of its component courses, otherwise it does not map). If more granularity is required, more quantitative or nuanced mappings are possible. The following schemes have been used successfully by early adopters of the ISCB competency framework to evaluate existing or new degree and multi-course programs:

0, 1, 2 mapping

For each course/competency pair, we give a score of 0 to a course that does not map to the competency, 1 to a course that maps to the competency incidentally and 2 to a course that focuses on the competency. While there may be grey areas in deciding whether an overlap is incidental or a focus, this mapping typically provides a coarse enough gradation to make such assignments straightforward. For example, an introductory molecular biology course in which the students have to create a poster and give a class presentation might get a score of 2 for the life sciences competency (Competency A3), a score of 1 for the Communication competency (Competency K3), and a score of 0 for the computing infrastructure competency (competency I3). We can then sum up the scores for each competency across all the courses to estimate the extent to which the whole program maps to this competency. Figure 4(a) provides an example of a 0,1,2 mapping.

Assessment weight mapping

This is only possible if the assessment components of each course are known, including how they map to the course learning outcomes, and we have a scheme assigning weights to the respective assessments. In this case, each competency gets a score for each course based on the proportion of the total assessment marks in the course dedicated to assessing this competency (as identified by overlapping competency KSAs and course learning outcomes). The scores for the entire program are obtained by adding up the scores across all component courses. Note that in most cases, competency mapping is used primarily as a tool for guiding reflection on, and improvement of, an existing program. While assessment weight mapping is the most comprehensive approach, it is also the most labour intensive and may not be needed except in the most formal evaluation cases, for example for degree accreditation.

In both cases, a heatmap representation can be used to visualise the mapping. Fig. 4(b) provides such an example for an assessment weight mapping.

A special case of such mapping is remapping a course or program that has previously been mapped to an older version of the framework. Working from an existing mapping can save one a great deal of effort. Changes in the competencies and their elaboration in KSAs can still make this a challenging task, however. We have provided some materials to facilitate understanding how the Version 2 and Version 3 competencies are related.

(a)

	ISCB Competency Version 3	Module 1	Module 2	Module n	Thesis project	Total
A3	Work at depth in at least one technical area aligned with the life sciences.	2				2
B3	Prepare life science data for computational analysis				2	2
C3	Have a positive impact on scientific discovery through bioinformatics	2				2
D3	Use data science methods suitable for the size and complexity of the data			2	2	4
E3	Manage own and others' data according to community standards and principles			1	2	3
F3	Make appropriate use of bioinformatics tools and resources				2	2
G3	Contribute effectively to the design and development of user-centric bioinformatics tools and resources		2			2
H3	Make appropriate and efficient use of scripting and programming languages		2		1	3
I3	Construct, manage and maintain bioinformatics computing infrastructure of varying complexity					0
J3	Comply with professional, ethical, legal and social standards and codes of conduct relevant to computational biology					0
K3	Communicate meaningfully with a range of audiences - within and beyond your profession				1	1
L3	Work effectively in teams to accomplish a common goal		1		1	2
M3	Engage in continuing professional development in bioinformatics				1	1

Key	Value
The module does not address the competency at all	0
The course addresses the competency but it is not its main focus (e.g. a programming course with a group project ad	1
The competency is the focus of the course, e.g. a course on python scripting addresses competency H3	2

(b)

CO → GC Mapping	Engineers Australia Stage 1 Competencies for Core+Elective Courses															
Courses (CO)	1.1	1.2	1.3	1.4	1.5	1.6	2.1	2.2	2.3	2.4	3.1	3.2	3.3	3.4	3.5	3.6
BABS1201	84	-	-	-	-	-	-	-	-	-	-	16	-	-	-	-
CHEM1011	92	-	-	-	-	-	-	4	-	-	1	1	-	1	1	1
CHEM1031	92	-	-	-	-	-	-	4	-	-	1	1	-	1	1	1
COMP1917	2	13	13	-	-	4	21	21	9	3	-	6	-	-	-	10
COMP1927	21	10	9	-	9	-	28	1	19	2	-	-	-	-	-	1
ENGG1000	-	-	7	-	-	-	6	-	6	17	11	10	10	3	11	18
MATH1081	21	58	-	-	-	-	-	-	-	-	-	21	-	-	-	-
MATH1131	28	65	-	-	-	-	-	-	-	-	-	7	-	-	-	-
MATH1141	28	65	-	-	-	-	-	-	-	-	-	7	-	-	-	-
MATH1231	28	65	-	-	-	-	-	-	-	-	-	7	-	-	-	-
MATH1241	28	65	-	-	-	-	-	-	-	-	-	7	-	-	-	-
PHYS1111	46	-	-	-	-	-	-	-	-	-	-	23	-	20	8	4
PHYS1121	100	-	-	-	-	-	-	-	-	-	-	-	-	-	-	-
PHYS1131	100	-	-	-	-	-	-	-	-	-	-	-	-	-	-	-
SENG1031	-	-	4	7	1	17	20	20	9	10	-	10	1	-	-	2

Figure 4: *Illustrations of mechanisms for mapping competencies to training programs. (a) Example of a generic 0,1,2 mapping of the competencies to a training program. (b) Example of a heatmap to visualise mapping of competencies to the curriculum. The image shows a heatmap used to visualise mapping of UNSW's bioinformatics engineering curriculum to the Engineers Australia Stage 1 competencies. Courtesy of Bruno Gaeta, UNSW Sydney, Australia. Heatmap software developed by John Shepherd, UNSW Sydney. For more information on this program and its relationship to an earlier version of the ISCB competencies, see (Mulder et al. 2018).*

4. Conclusion

While we hope this document will be a helpful guide for other educators in the field, we note that it is not intended to be the first or the last word on the topic. Educators would do well to learn more about competency-based education and training than we have room to cover explicitly here. We have provided some basic references, but practices are continually evolving and practitioners would be well advised to keep current with best practices in the field as they develop. The competencies themselves can also be considered a living document. Since their first conception, the competencies have been substantially revised twice in response to extensive community input. We can reasonably expect they will continue to evolve with experience in their use and with the changing landscape of the subject matter. Computational biology education has changed dramatically as it has come to be recognized as not just a specialty of a few expert interdisciplinary researchers, but rather an essential part of the knowledge base for any modern work in biomedical science or its applications. At the same time, the educational world has been forced by the COVID-19 pandemic into a dramatic shift towards increasing use of online and technology-assisted education, which has challenged our notions of how to teach and assess learning. While the pandemic may be moving towards resolution, educational practice is unlikely ever to return fully to what it was before. We hope that as our notion of the need for bioinformatics competencies and how to use them continues to evolve, so too will our experience with them and the guidance our community can provide to one another.

References

Burke, John., ed. 2005. *Competency Based Education And Training*. Routledge. ISBN 1135387893, 9781135387891

Campbell, Chad E., and Ross H. Nehm. 2013. "A Critical Analysis of Assessment Quality in Genomics and Bioinformatics Education Research." *CBE—Life Sciences* Education. https://doi.org/10.1187/cbe.12-06-0073.

Davies, Alan, Julia Mueller, and Georgina Moulton. 2020. "Core Competencies for Clinical Informaticians: A Systematic Review." *International Journal of Medical Informatics* 141 (September): 104237.

Magana, Alejandra J., Manaz Taleyarkhan, Daniela Rivera Alvarado, Michael Kane, John Springer, and Kari Clase. 2014. "A Survey of Scholarly Literature Describing the Field of Bioinformatics Education and Bioinformatics Educational Research." *CBE Life Sciences Education* 13 (4): 607–23.

Mulder, Nicola, Russell Schwartz, Michelle D. Brazas, Cath Brooksbank, Bruno Gaeta, Sarah L. Morgan, Mark A. Pauley, et al. 2018. "The Development and Application of Bioinformatics Core Competencies to Improve Bioinformatics Training and Education." *PLOS Computational Biology*. https://doi.org/10.1371/journal.pcbi.1005772.

Nicholls, Gill. 2002. "Developing Teaching and Learning in Higher Education." https://doi.org/10.4324/9780203469231.

Welch, Lonnie, Fran Lewitter, Russell Schwartz, Cath Brooksbank, Predrag Radivojac, Bruno Gaeta, and Maria Victoria Schneider. 2014. "Bioinformatics Curriculum

Guidelines: Toward a Definition of Core Competencies.” *PLoS Computational Biology*. https://doi.org/10.1371/journal.pcbi.1003496.
Welch, Lonnie R., Russell Schwartz, and Fran Lewitter. 2012. “A Report of the Curriculum Task Force of the ISCB Education Committee.” *PLoS Computational Biology* 8 (6): e1002570.
Wilkinson, Mark D., Michel Dumontier, I. Jsbrand Jan Aalbersberg, Gabrielle Appleton, Myles Axton, Arie Baak, Niklas Blomberg, et al. 2016. “The FAIR Guiding Principles for Scientific Data Management and Stewardship.” *Scientific Data* 3 (March): 160018.
Wilson Sayres, Melissa A., Charles Hauser, Michael Sierk, Srebrenka Robic, Anne G. Rosenwald, Todd M. Smith, Eric W. Triplett, et al. 2018. “Bioinformatics Core Competencies for Undergraduate Life Sciences Education.” *PloS One* 13 (6): e0196878.

Appendix

A1. Related competency frameworks

In understanding the ISCB competency framework, it can be helpful to know about some related frameworks. Here we provide links to a few competency frameworks that have specifically helped to inform the ISCB's approach:

- A clinical bioinformatics competency framework to support Health Education England to prepare clinical practitioners for the application of genomics in the healthcare service.
- The RItrain competency framework for managers and leaders of research infrastructure.
- The CORBEL competency framework for technical operators of research infrastructure
- The BioExcel competency framework for scientists working on biomolecular modelling and simulation.
- The Safety Sciences competency profile developed by imi-train
- Specialist in Medicines Development – developed by PharmaTrain and IFAPP
- Researcher Development Framework (all disciplines) – developed by Vitae, which includes a 'lens' for Employability competencies
- Competency areas for Medical Information Professionals – developed by The Pharmaceutical Information and Pharmacovigilance Association (PIPA)
- Guidelines for the European Registration of Toxicologists – developed by EUROTOX
- Professional Registration offered by the Science Council
- UK NOS: Bioinformatics and Life Sciences.
- UK level 7 apprenticeship standard.
